# Gabapentin drug interactions in water and aqueous solutions of green betaine based compounds through volumetric, viscometric and interfacial properties

**DOI:** 10.1038/s41598-025-99596-3

**Published:** 2025-05-14

**Authors:** Elaheh Janbezar, Hemayat Shekaari, Mohammad Bagheri

**Affiliations:** Department of Physical Chemistry, Faculty of Chemistry, University of Tabriz, Tabriz, 5166616471 Iran

**Keywords:** Betaine, Gabapentin, Critical micelle concentration, Density, Viscosity *B*-coefficient, Surface tension, COSMO and thermophysical properties, Chemistry, Ionic liquids

## Abstract

Betaine as a bio-based surfac﻿tant, has been found in a variety of natural sources. Betaine improves drug absorption, protect drugs from degradation, and enhance the performance of various therapeutic and hygiene products. To investigate the interactions between gabapentin (an antiepileptic drug) and betaine-based compounds, series of experiments were conducted at 298 K. These experiments involved volumetric, viscometric, and surface tension measurements of aqueous solutions containing gabapentin and various betaine-based compounds, including betaine, betaine octyl ester chloride ionic liquid and betaine-urea deep eutectic solvent (molar ratio of 1:2). Additionally, the Conductor like Screening Model (COSMO) method were employed to gain further insights into molecular interactions governing these systems. The volumetric studies revealed that the standard partial molar volumes *V*^0^_φ_ of the betaine-based compounds increased with increasing gabapentin concentration, suggesting significant solute-solvent interactions. The apparent specific volume (ASV) and the hydration number (*n*_H_) for gabapentin in the examined systems were calculated. The analysis of the obtained ASV and *n*_H_ values indicated that gabapentin exhibits a bitter taste in aqueous deep eutectic solvent (DES) solutions and in the presence of betaine it gets most dehydrated. The viscosity measurements, analyzed using the Jones-Dole equation, yielded negative viscosity *B*-coefficient values for the betaine octyl ester chloride ionic liquid, suggesting its potential to enhance the drug-related properties of gabapentin. Surface tension measurements were used to determine the critical micelle concentration (CMC) of the betaine-based compounds and their related surface properties such as interface surface pressure ($\Pi$), and Gibbs maximum excess surface concentration (${\Gamma _{\hbox{max} }}$). The CMC values decreased with increasing gabapentin concentration, indicating enhanced micellization. The betaine octyl ester chloride ionic liquid exhibited the lowest CMC, suggesting its superior ability to form micelles. The results of this study suggested that the betaine-based compounds improve drug absorption, protect drugs from degradation, and enhance the performance of various therapeutic and hygiene products underscores their importance in both the pharmaceutical and industrial sectorsunds, particularly the betaine octyl ester chloride, may have the potential to improve the drug-related properties of gabapentin.

**Supplementary Information:**

The online version contains supplementary material available at 10.1038/s41598-025-99596-3.

## Introduction

Surfactants are essential compounds in a wide range of industrial and pharmaceutical applications, functioning primarily as emulsifying agents. Surfactants represent indispensable agents extensively applied across a broad range of industrial and pharmaceutical fields, predominantly serving as emulsifying agents. Among their most significant applications is their involvement in the formulation and stabilization of nanofluids. In pharmaceutical applications, these nanofluids are utilized as highly effective carriers for targeted drug delivery, with their physicochemical stability and performance being critically dependent on the presence and action of surfactants^1,2^. These substances enable the integration of immiscible phases, such as hydrophobic and hydrophilic components, by reducing surface tension, thereby enhancing the solubility and bioavailability of compounds that are otherwise poorly soluble in water^3^. In pharmaceutical formulations, surfactants play a critical role in enhancing the absorption of therapeutic agents, particularly those with low water solubility, by facilitating their dissolution in biological fluids^4^. This is particularly important in the gastrointestinal tract, where surfactants improve the penetration and absorption of drugs, ensuring a larger fraction reaches systemic circulation^5^. Furthermore, surfactants contribute to the stability and dispersion of pharmaceutical formulations, allowing for a more uniform distribution of the drug throughout the body, which is vital for optimizing therapeutic outcomes^6^. One of the key surfactants used in various applications is betaine, a naturally occurring compound derived from glycine^7^. Betaine, a trimethylglycine compound found in various foods, has garnered attention for its diverse pharmaceutical applications. It exhibits antioxidant, neuroprotective, and anti-inflammatory properties, potentially benefiting conditions like neurodegenerative diseases and obesity^8^. Betaine is highly soluble in aqueous environments and exhibits surface-active properties that make it an effective emulsifier and surfactant in both pharmaceutical and industrial formulations^9^. It is particularly advantageous in lipid-based drug delivery systems, where its ability to integrate oil and water-based components is crucial. In addition to its emulsifying role, betaine helps protect pharmaceuticals from degradation in harsh conditions, such as the acidic environment of the stomach, thereby enhancing the stability and efficacy of the drug^10^. Betaine’s biocompatibility and moisturizing properties also make it a popular ingredient in cosmetics and hygiene products, where it contributes to foam formation and enhances the perceived cleanliness and texture of these products^11^. In addition to its role in drug delivery systems, betaine offers several other benefits due to its chemical properties. As a biobased surfactant, it aligns with principles of green chemistry by reducing reliance on synthetic chemicals and minimizing environmental impact^12^. Betaine is found in a variety of natural sources, including beetroots, spinach, cereals, marine organisms, and animal tissues^13^. Its natural origin, combined with its ability to reduce surface tension and promote the distribution of substances within solutions, further highlights its versatility and sustainability as an emulsifier^14^. Furthermore, betaine demonstrates both thermal and chemical stability, allowing it to be applied across a wide range of conditions, further reinforcing its role as a multifunctional agent in both pharmaceutical and cosmetic formulations^15^. Surfactants, like betaine, are indispensable in enhancing the efficacy, stability, and bioavailability of pharmaceutical formulations^7^. Their ability to improve drug absorption, protect drugs from degradation, and enhance the performance of various therapeutic and hygiene products underscores their importance in both the pharmaceutical and industrial sectors^16^. By promoting the solubility, dispersion, and stability of active ingredients, surfactants such as betaine are crucial for optimizing product quality, therapeutic outcomes, and consumer experience across a variety of applications^6^.

The surface activity characteristics exhibited by the betaine-based compounds, have led to the determination of their critical micelle concentration (CMC), a crucial criterion for monitoring their behavior in aqueous solutions^17^. As such, this property has been applied in the processing of pharmaceuticals, particularly in the investigation of load and release properties of various drugs and can be used as an agent to improve drug adsorption and penetration through cell membrane^18^. Accordingly, introducing new biocompatible betaine-based compounds could help green processing of different drugs. Additionally, betaine-based compounds can enhance hydration in aqueous solutions, leading to improved drug dissolution and absorption, thereby further improving therapeutic outcomes for patients^19^. Incorporating betaine-based compounds in drug processing presents a promising strategy for enhancing drug efficiency, reducing overall drug consumption, and ultimately improving therapeutic outcomes for patients. By leveraging the unique solubility-enhancing and stabilizing properties of these compounds, formulations can be optimized to ensure better drug absorption and bioavailability, leading to more effective treatments with lower dosages^20^. One of the most reliable methods for the CMC point determination is the utilization of the static surface tension measurements by employing Wilhelmy plate (PL22) with different approaches to the phenomenon that could provide more information on interfacial behavior influenced by micelle formation^21^.

Interfacial electron density is another approach to the surface characteristics of a molecule that could be achieved by DFT calculations^22^. A simple and practical DFT calculation is provided by Dmol^3^ named conductor like screening model (COSMO) that provides the surface cavity, volume solvation energy, and *σ*-profile as dielectric characteristics of the chemical structure^23^. Accordingly, it could provide other DFT-based properties that could help to interpret the observed macroscopic results with different microscopic approach^24^. The* σ*-profile of a molecule provides substantial information about the electrostatic distribution on the molecule structure^25^. Therefore, DFT calculations provides another microscopic approach to the phenomenological aspect of CMC and molecular structure^26^.

**Fig. 1:**
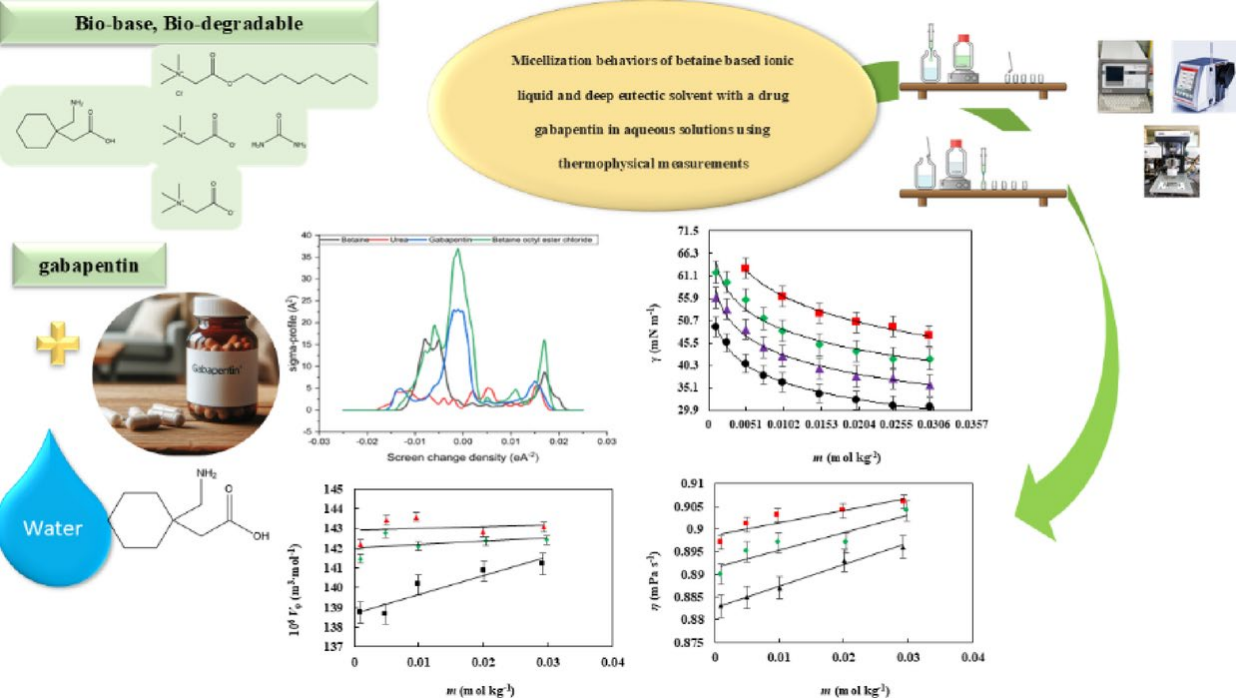
Micellization behavior of betaine-based ionic liquids and deep eutectic solvents with gabapentin in aqueous solutions, analyzed through thermophysical measurements and experimental techniques.

This study delves into the thermodynamic behavior of gabapentin (GBP) in the presence of betaine-based compounds, including betaine, betaine octyl ester chloride ionic liquids (ILs), and betaine-based deep eutectic solvents (DESs), across various concentration ranges. The primary objective is to investigate the potential of these betaine-based compounds to enhance the drug related properties of GBP, particularly within the gastrointestinal tract. To achieve this, a comprehensive experimental approach involving volumetric (density), viscosity, and static surface tension measurements was employed. The viscosity measurements yielded viscosity *B*-coefficients for systems containing GBP in aqueous solutions of betaine-based compounds at molal concentrations of 0.01, 0.03, and 0.05 mol kg⁻¹. Surface tension measurements were used to determine critical micelle concentration (CMC), standard free energy of micellization (*ΔG*_mic_), standard Gibbs free energy of adsorption ($\Delta G_{{ad}}^{0}$), surface pressure ($\Pi$), minimum surface area occupied per molecule (*A*_min_), and Gibbs maximum excess surface concentration (*Γ*_max_). To gain deeper insights into the intermolecular interactions within the studied systems, a computational approach involving the conductor-like screening model (COSMO) was utilized^27^. Density functional theory (DFT) calculations based on Dmol3 and COSMO results were performed to provide a microscopic perspective on the CMC phenomenon for the betaine-based compounds in the presence of GBP. The collective findings of this study have significant implications for the pharmaceutical industry (Fig. 1).

## Experimental

### Chemicals

The specification of the utilized chemicals has been provided within the Table 1. The table discusses the chemical name, chemical formula, origin (provenance), CAS registry number (CAS.no), molar mass, and mass fraction (purity) of the utilized chemicals. The deep eutectic solvent (DES) was prepared by combining betaine and urea in a 1:2 molar ratio. The mixture was heated to 80 °C with vigorous agitation for 2 h. The schematic representation of the DES synthesis route has been provided in Fig S1 of the supporting Information.

**Table 1:** The specification of the utilized chemicals.

Chemical name	Chemical Formula	Origin	CAS.no	Molar mass (g ⋅mol^−1^)	Mass fraction (purity)
Betaine	C_5_H_11_NO_2_	Merck	107-43-7	117.148	> 99%
Gabapentin	C_9_H_17_NO_2_	Merck	60142-96-3	171.237	> 99%
Urea	NH_2_CONH_2_	Merck	57-13-6	60.06	> 99%
1-Chlorooctane	C_8_H_17_Cl	Merck	111-85-3	148.67	> 99%
Acetonitrile	CH_3_CN	Merck	75-05-8	41.05	> 99%
Diethyl ether	C_4_H_10_O	Merck	60-29-7	74.12	> 99%

All of the utilized chemical were used without further purification.

### Synthesis of ionic liquid

The synthesis of betaine octyl ester chloride ionic liquid was initiated by combining betaine and 1-chlorooctane in a 1:1.2 molar ratio within a 250 mL round-bottom flask (presented schematically within Fig S2). Acetonitrile (50 mL) was employed as the reaction solvent to facilitate the reaction kinetics. The reaction mixture was subjected to reflux under an inert argon atmosphere at 353.15 K for 72 h, with vigorous magnetic stirring. Upon completion of the reaction, the ionic liquid was purified to remove residual solvent and unreacted alkyl halide. This purification process involved a combination of distillation and vacuum treatment using a rotary evaporator. Distillation was continued until a solid powder was obtained. Subsequently, diethyl ether was added to precipitate the product and further eliminate any unreacted alkyl halide. The solid precipitate was then dried under vacuum to remove residual diethyl ether^28^. The synthesized ionic liquid was characterized using Fourier Transform Infrared (FT-IR) and Nuclear Magnetic Resonance (NMR) spectroscopy^29^. The corresponding IR and ¹H-NMR spectra, provided in the supporting information (Figures S3 and S4), confirmed the successful synthesis of the ionic liquid and indicated a high purity of over 98%.

### Synthesis route of DES (betaine-urea)

The synthesis of the deep eutectic solvent (DES) was carried out through a systematic and controlled procedure to ensure high purity and reproducibility. Initially, betaine and urea were accurately weighed in a 1:2 molar ratio and transferred into a dry, clean, 250 mL round**-**bottom flask to prevent contamination. The reaction mixture was then subjected to continuous magnetic stirring at an elevated temperature of 353.15 K (80 °C) for a duration of 2 h, ensuring complete dissolution and homogeneous mixing of the components^30^. The stirring process facilitated the formation of strong hydrogen bonding interactions between betaine and urea, which are essential for the formation of a stable eutectic system. Upon completion of the reaction, the resulting DES was allowed to cool leading to the formation of a solid product. The final product was collected, stored in an airtight container to prevent moisture absorption, and utilized without further purification. This synthesis approach ensured the successful preparation of DES with high stability and desired physicochemical properties, making it suitable for subsequent experimental applications^31,32^.

The characterization of the prepared DES was carried through FT-NMR spectroscopy (Shown in Fig S5), through the analysis of the illustrated peaks it can be concluded that the ¹H NMR spectrum of the betaine-urea (1:2) deep eutectic solvent exhibits several key features that reflect the interactions between the two compounds. The spectrum shows chemical shifts consistent with both hydrogen bonding and coulombic interactions between betaine and urea. The urea amide protons typically appear in the range of 5.0–7.0 ppm, where they are downfield shifted due to hydrogen bonding with betaine’s carboxylate group. These shifts provide evidence for the strong inter-ionic interactions that contribute to the stability of the DES. The trimethylammonium group (-N(CH₃) ₃) of betaine shows a sharp singlet around 3.0-3.5 ppm, typical of a quaternary ammonium environment^33^. Additionally, the methylene (-CH₂-) protons adjacent to the carboxylate and ammonium groups in betaine appear between 3.5 and 4.5 ppm, where they are deshielded due to interactions with urea. This confirms the hydrogen bonding between betaine and urea in the system. The total number of protons in the system, considering the 1:2 molar ratio of betaine to urea, is consistent with the expected number of protons for the two components, further supporting the structure of the DES. These observations highlight the significant role of both hydrogen bonding and Coulombic forces in the formation and stabilization of the deep eutectic solvent^31^.

### Instrumentation

#### Surface tension measurement

The surface tension of aqueous solutions containing betaine compounds (betaine, DES and the ionic liquid, betaine octyl ester chloride) and varying concentrations of gabapentin (0.0000–0.0500 mol kg⁻¹) was measured at a constant temperature of 298 K using a KRÜSS Easy Dyne K20 tensiometer (Germany) equipped with a Wilhelmy plate (PL22). The instrument’s uncertainty in measuring surface tension was estimated to be ± 0.01 mN m^−1^. To ensure accurate measurements, the Wilhelmy plate was rigorously cleaned before each experiment. The cleaning process involved rinsing with ultrapure, double-distilled, deionized water, followed by high-purity acetone (specifications provided in Table 1). Subsequently, the plate was heated to a red-hot state. The critical micelle concentration (CMC) of the betaine compounds was determined by extrapolating the inflection point observed in the surface tension versus molality plot.

#### Volumetric measurement

The solutions of gabapentin in various concentrations of aqueous betaine-based compounds (betaine, betaine: urea deep eutectic solvent, betaine octyl ester chloride ionic liquid) were prepared using an analytical balance (AND, GR 202) with a resolution of 10⁻⁵ kg and a precision of 2 × 10⁻⁴ kg. The density of these solutions was measured using a digital vibrating U-shaped densitometer (KYOTO ELECTRONICS DA210) with a precision of 10⁻^9^ g cm⁻³. The density measurements were conducted at atmospheric pressure (0.087 MPa). The uncertainty in density measurements was estimated to be ± 4 × 10⁻⁵ g cm⁻³. The instrument was calibrated using distilled water (air-water program). The combined standard uncertainty of density was determined according to NIST standards, resulting in a value of 1 kg m⁻³.

#### Viscosity measurement

The viscosity of the solutions was determined using an Anton Paar Rolling-ball viscometer, Lovis 2000 M/ME. The temperature was precisely controlled to ± 0.005 K by an integrated Peltier thermostat. The measurement principle of the Lovis 2000 M/ME is based on the falling ball method. A calibrated glass capillary, supplied by the manufacturer with the instrument, was filled with the sample solution. The falling time of a steel ball within the capillary was measured to approximate both kinematic and dynamic viscosities. The capillary was calibrated by the manufacturer using viscosity standard fluids. The combined uncertainty of the viscosity measurements was 0.001 mPa s^−1^.

## Results and discussion

### Volumetric results

The study investigated the effect of three betaine compounds (betaine, DES, and betaine octyl ester chloride) on the density of gabapentin solutions. The experimental densities (*ρ*) of gabapentin in water and varying concentrations (0.01, 0.03, and 0.05 mol kg⁻¹) of aqueous solutions containing these betaine compounds were determined as a function of gabapentin molality (m) at a constant temperature of 298 K and has been tabulated within the Table 2. The results revealed a positive correlation between the density of the examined solutions and the content of betaine-based compounds. In other words, the densities of both (gabapentin + water) and (gabapentin + water + betaine-based compounds) solutions increased with increasing concentrations of the betaine compounds.

**Table 2:** The density (*ρ*), and apparent molar volume (${V_\varphi }$), of GBP in aqueous Betaine, DES, and betaine octyl ester chloride in 0.01,0.03,0.05 molality concentration under 298 K.

^a^*m*_solvent_ (mol kg^−1^)								
0.0102			0.0306			0.0498		
Betaine								
^b^ *m*_solution_ (mol kg^−1^)	10^−3^ *ρ* (kg cm^−3^)	10^−6^ *V*_φ_ (m^3^ mol^−1^)	^b^ *m*_solution_ (mol kg^−1^)	10^−3^ *ρ* (kg cm^−3^)	10^−6^ *V*_φ_ (m^3^ mol^−1^)	^b^ *m*_solution_ (mol kg^−1^)	10^−3^ *ρ* (kg cm^−3^)	10^−6^ *V*_φ_ (m^3^ mol^−1^)
0.0000	0.99819	-	0.0000	0.99870	-	0.0000	0.99900	-
0.0010	0.99822	138.747	0.0010	0.99870	141.474	0.001	0.99903	141.193
0.0049	0.99834	140.697	0.0049	0.99881	142.774	0.005	0.99914	143.212
0.0100	0.99850	140.154	0.0099	0.99896	142.115	0.0097	0.99927	143.491
0.0201	0.99880	140.851	0.0204	0.99926	142.372	0.02	0.99957	142.774
0.0293	0.99907	141.21	0.0298	0.99953	142.427	0.0294	0.99983	143.048
DES (Betaine: Urea 1:2)								
0.0000	0.99799	-	0.0000	0.99897	-	0.0000	1.00005	-
0.0019	0.99817	140.862	0.0010	0.99901	142.559	0.0010	1.00009	142.356
0.0050	0.99846	142.837	0.0050	0.99917	142.225	0.0050	1.00025	142.379
0.0100	0.99894	142.676	0.0099	0.99936	142.777	0.0099	1.00043	143.470
0.0200	0.99987	143.13	0.0200	0.99975	143.141	0.0193	1.00079	143.504
0.0295	1.00075	143.448	0.0296	1.0001	143.644	0.0298	1.00117	143.911
IL (betaine octyl ester chloride)								
0.0000	0.99896	-	0.0000	0.99961	-	0.0000	1.00007	-
0.0010	0.99899	140.271	0.0010	0.99964	140.274	0.0010	1.00010	140.790
0.0050	0.99911	141.349	0.0049	0.99975	142.776	0.0049	1.00021	142.518
0.0099	0.99926	141.155	0.0099	0.99989	142.913	0.0100	1.00034	144.090
0.0198	0.99955	141.527	0.0203	1.00018	143.115	0.0199	1.00062	143.493
0.0301	0.99984	141.905	0.0299	1.00044	143.396	0.0289	1.00085	144.151

The standard uncertainties for molality, temperature and pressure were *u* (*m*) *=* 0.001 mol kg^−1^, *u* (*T*) *=* 0.2 K, *u* (*P*) *=* 10.5 hPa, respectively with level of confidence 0.95. The standard combined uncertainty for density and apparent molar volume were about, *u*_*c*_ (*ρ*) *=* 0.06 × 10^−3^ g cm^−3^ and *u*_*c*_(*V*_*φ*_) = 5 × 10^−5^m^5^ mol^−1^ (level of confidence 0.68), respectively. ^a^The molality of the prepared betaine compound in water (solvent). ^b^The molality of the prepared gabapentin in aqueous betaine-based compounds.

**Fig. 2:**
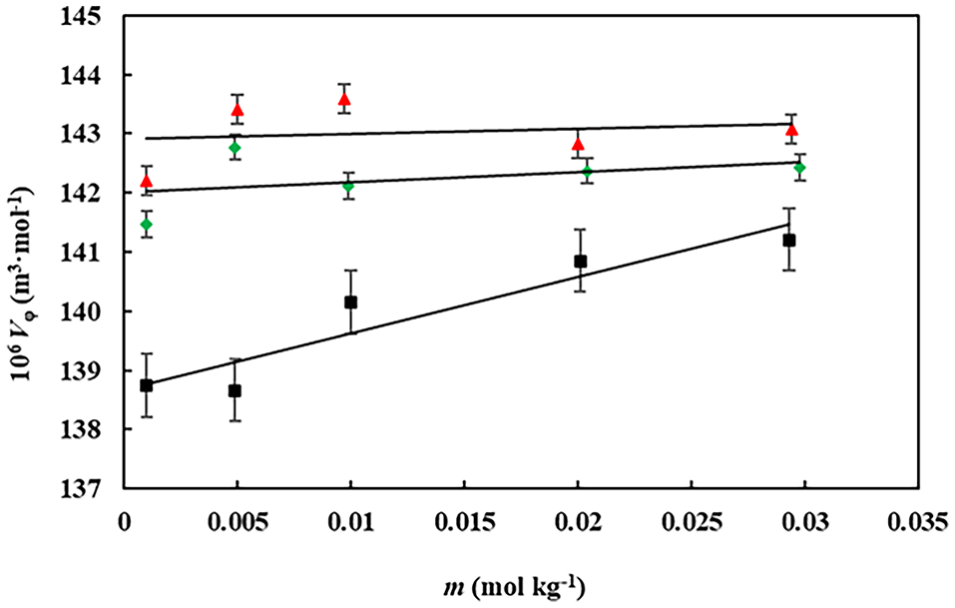
The apparent molar volumes ${V_\varphi }$(m^5^ mol^−1^) of gabapentin versus its molality, *m* (mol kg^−1^) in aqueous betaine solutions with varying molalities: ▲, 0.0500; ◆, 0.0300; ▪, 0.0100 at *T* = 298 K.

The apparent molar volumes (${V_\varphi }$) of gabapentin in the solutions under study were computed using the following expression^34^:

1 $${V_\varphi }=\frac{M}{\rho } - \frac{{(\rho - {\rho _0})}}{{m\rho {\rho _0}}}$$

where *M* is the molar mass of gabapentin, *m* denotes the molality of gabapentin in aqueous betaine-based compound solutions, and $~\rho$, and $~{\rho _0}$ represent the densities of (gabapentin + water + betaine-based compounds) and (water + betaine-based compounds) solutions, respectively. The apparent molar volume (${V_\varphi }$) values of gabapentin as a function of its molality in aqueous betaine solutions has been depicted within Table 2. At the studied temperature, the values of increased with increasing concentrations of the betaine compound (as illustrated in Fig. 2).

A strong linear correlation was observed between the ${V_\varphi }$ values and gabapentin molality (*m*). Consequently, apparent molar volumes at infinite dilution (standard partial molar volume,$V_{\varphi }^{0}$) values can be determined through utilization of least-squares fitting to Masson’s equation^35^:

2 $${V_\varphi }={V_\varphi }^{0}+{S_v}m$$

here *S*_*v*_ represents the empirical parameters. Given the negligible nature of solute-solute interactions at infinite dilution, standard partial molar volumes $V_{\varphi }^{0}$offer crucial information regarding solute-solvent interactions^36^. The values of ${V_\varphi }^{0}$and *S*_*v*_ together with their standards deviation of the ${V_\varphi }^{0}$ values are reported in Table 3.

**Table 3:** Standard partial molar volumes ($~V_{\varphi }^{0}~$), adjustable parameter of Eq. 2 ($~{S_v}~$) and standard deviations ($\sigma (~v_{\varphi }^{0}~)$) for GBP in aqueous solutions of betaine-based compounds at 298 K.^a^

^a^ *m* (mol kg^−1^)	10^6^ $~V_{\varphi }^{0}~$ (m^3^ mol^−1^)	10^6^*S*_v_ (m^3^ mol^−2^kg)	($\sigma (~V_{\varphi }^{0}~)$)
Betaine			
0.0098	138.677 ± 0.050	64.835 ± 0.300	0.04
0.0297	142.010 ± 0.015	31.385 ±0. 631	0.02
0.0501	142.916 ± 0.040	8.232 ± 0.800	0.09
DES (betaine-urea 1:2)			
0.0103	141.695 ± 0.054	67.432 ±0. 333	0.07
0.0299	142.292 ± 0.0751	31.835 ± 0.160	0.10
0.0501	142.407 ± 0.030	39.798 ± 0.894	0.04
IL (betaine octyl ester chloride)			
0.0103	140.664 ± 0.070	43.886 ± 0.796	0.01
0.0294	141.481 ± 0.093	76.856 ± 0.213	0.03
0.0496	141.791 ± 0.137	93.956 ± 0.931	0.08

The standard uncertainties for molality, temperature and pressure were *u* (*m*) *=* 0.001 mol kg^−1^, *u* (*T*) *=* 0.2 K, *u* (*P*) *=* 10.5 hPa, respectively with level of confidence 0.95. The standard combined uncertainty for density and apparent molar volume were about, *u*_*c*_ (*ρ*) *=* 0.06 × 10^−3^ g cm^−3^ and *u*_*c*_(*V*_*φ*_) = 5 × 10^−5^ m^3^ mol^−1^ (level of confidence 0.68), respectively.

^a^The molality of the prepared betaine compound in water (solvent).

The data presented in Table 3 reveals changes in the standard partial molar volume (${V_\varphi }^{0}$) and related parameters for gabapentin (GBP) in various solvent systems, including aqueous betaine, DES (betaine-urea 1:2), and betaine octyl ester chloride (IL), across different concentrations. The ${V_\varphi }^{0}$ as suggested by its meaning refers to the change in volume of the solute (GBP in this study) when it gets firstly added to the solution. The decrease in ${V_\varphi }^{0}$ values, suggests that attractive solvent-solvent interaction is favorable, and if the values of ${V_\varphi }^{0}$increased, it indicates that the repulsive solvent-solute interaction are more favorable^37^.

For the betaine system, the ${V_\varphi }^{0}$increases with concentration, particularly at higher concentrations, where it reaches 142.916 m³ mol⁻¹ at 0.0501 mol kg⁻¹. This suggests that higher concentrations of betaine facilitate stronger solute-solvent interactions, allowing more accommodation of GBP molecules in the solvent^38^. Similarly, in the DES system, the ${V_\varphi }^{0}$ values also rise with increasing concentration, but the increase is more modest compared to the betaine system. At 0.0103 mol kg⁻¹, the ${V_\varphi }^{0}$ is 141.695 m³ mol⁻¹, and it increases to 142.407 m³ mol⁻¹ at 0.0501 mol kg⁻¹. This indicates that the hydrogen bonding interactions between betaine, urea, and water in the DES system limit the extent to which GBP molecules can be accommodated, leading to a less pronounced increase in ${V_\varphi }^{0}$values^39^. For the IL system, the ${V_\varphi }^{0}$ increases as well, with a notable increment at intermediate concentrations. At 0.0294 mol kg⁻¹, the ${V_\varphi }^{0}$ is 141.481 m³ mol⁻¹, and it rises to 141.791 m³ mol⁻¹ at 0.0496 mol kg⁻¹. This suggests that ILs provide a relatively favorable environment for GBP molecules, though not as much as aqueous betaine solutions.

These observations suggest that GBP interacts most favorably with aqueous betaine, where the solvent structure is most disrupted, providing more space for the solute molecules to interact with the solvent^40^. On the other hand, DES systems, with their strong hydrogen bonding interactions, exhibit a more rigid solvent structure, reducing the ability of GBP to disrupt this structure and resulting in a smaller increase in ${V_\varphi }^{0}$values^41^. The IL system falls in between, showing an increase in ${V_\varphi }^{0}$ values with concentration but to a lesser extent than the betaine system. Overall, the data suggest that GBP shows the most favorable interactions with betaine, followed by ILs and DES, respectively.

The ${V_\varphi }^{0}$ of GBP, a key parameter for understanding solute-solvent interactions, was positive across all concentrations of aqueous betaine, betaine octyl ester chloride, and DES solutions. The increase in ${V_\varphi }^{0}$values with the rising concentrations of these cosolvents can be attributed to a synergistic effect of these cosolvents on the solvent structure^42^. Specifically, the presence of cosolvents disrupts the electrostriction of water molecules, leading to a more open solvent structure^43^. As the concentration of these cosolvents increases, this disruption intensifies, providing more space for GBP molecules to accommodate. This, in turn, enhances the solute-solvent interactions, contributing to the observed increase in ${V_\varphi }^{0}$values^44^. In contrast, a more modest increase in ${V_\varphi }^{0}$ values were observed with increasing concentrations of DES. This can be explained by the strong hydrogen bonding interactions between the DES components (betaine and urea) and water molecules, which results in a more structured solvent. As the concentration of DES increases, the solvent structure becomes more rigid, which limits the accommodation of GBP molecules and leads to a less pronounced increase in ${V_\varphi }^{0}$values. These findings underscore the differential effects of the solvent environment on the behavior of GBP in solution. In aqueous betaine systems, where the solvent structure is more disrupted, GBP can more easily interact with the solvent, leading to greater solute-solvent interactions and larger ${V_\varphi }^{0}$ values. In DES solutions, however, the stronger hydrogen bonding interactions between DES components and water restrict GBP’s ability to disrupt the solvent structure, resulting in a smaller increase in ${V_\varphi }^{0}$ values. This suggests that GBP may exhibit the most favorable interactions with betaine, followed by ILs and DES.

### Taste behavioral results

The investigation focused on the taste behavior of GBP when exposed to water and solutions containing aqueous betaine-based compounds, including betaine, deep eutectic solvents (DES), and ionic liquids (IL). This study utilized apparent specific volumes (*ASV*) to analyze the interactions within varying concentrations of these aqueous solutions. The research was conducted through a systematic application of a specific equation designed to quantify these relationships^45^:

3 $$ASV=\frac{{{V_\varphi }}}{M}$$

where *M* is the molar mass of *GBP*. The *ASV* values of GBP in both pure water and aqueous betaine-based compounds solution (Table 4) suggest that the addition of the studied betaine based-compounds does not significantly alter the physical properties related to the taste behavior of *GBP *^45^.

**Table 4:** The values of apparent specific volume (*ASV*) values for *GBP* in water and aqueous betaine-based compounds solutions at 298 K.

GBP in aqueous solutions of Betaine					
0.0100		0.0300		0.0500	
*m *(mol·kg^−1^)	ASV (cm^3^·g^−1^)	*m* (mol·kg^−1^)	ASV (cm^3^·g^−1^)	*m* (mol·kg^−1^)	ASV (cm^3^·g^−1^)
0.0000	-	0.0000	-	0.0000	-
0.0010	0.810	0.0010	0.826	0.0010	0.825
0.0049	0.822	0.0049	0.834	0.0050	0.836
0.0100	0.818	0.0099	0.830	0.0097	0.838
0.0201	0.823	0.0204	0.831	0.0200	0.834
0.0293	0.825	0.0298	0.832	0.0294	0.835

GBP in aqueous solutions of DES (betaine-urea 1:2)					
0.0100		0.0300		0.0500	
*m* (mol·kg^−1^)	ASV (cm^3^·g^−1^)	*m* (mol·kg^−1^)	ASV (cm^3^·g^−1^)	*m* (mol·kg^−1^)	ASV (cm^3^·g^−1^)
0.0000	-	0.0000	-	0.0000	-
0.0019	0.834	0.0010	0.833	0.0010	0.831
0.0050	0.833	0.0050	0.831	0.0050	0.831
0.0100	0.836	0.0099	0.834	0.0099	0.838
0.0200	0.838	0.0200	0.836	0.0193	0.838
0.0295	0.823	0.0296	0.839	0.0298	0.840

GBP in aqueous solutions of IL (betaine octyl ester chloride)					
0.0100		0.0300		0.0500	
*m* (mol·kg^−1^)	ASV (cm^3^·g^−1^)	*m* (mol·kg^−1^)	ASV (cm^3^·g^−1^)	*m* (mol·kg^−1^)	ASV (cm^3^·g^−1^)
0.0000	-	0.0000	-	0.0000	-
0.0010	0.819	0.0010	0.819	0.0010	0.822
0.0050	0.825	0.0049	0.834	0.0049	0.832
0.0099	0.824	0.0099	0.835	0.0100	0.841
0.0198	0.826	0.0203	0.836	0.0199	0.838
0.0301	0.829	0.0299	0.837	0.0289	0.842

The *ASV*value has been recognized in the literature as a significant criterion for assessing sweetness^46^. Research conducted by Birch et al. and Shekaari et al. indicates that an *ASV* value of approximately 0.33 correlates with a salty taste, while an *ASV* value around 0.52 is typically associated with a sour flavor. Moreover, an *ASV*value of 0.72 suggests that the substance is likely to possess a sweet taste, whereas values of 0.93 and above are indicative of a bitter taste^47,48^. Consequently, the acceptable range for sweetness is delineated as 0.5 < *ASV*< 0.7, which is considered the optimal range for sweetness perception^49^. In the context of the current study, as illustrated in Table 4, the *ASV*values observed fall within the range of 0.810–0.840 cm³ g⁻¹. This finding implies that gabapentin, despite being associated with a bitter taste according to the studies by Rao et al., exhibits a pronounced bitterness when combined with betaine-based compounds^50^. Specifically, the DES analyzed in this study demonstrate *ASV* values that are somewhat higher than those of both betaine and IL. This suggests that the taste of gabapentin is expected to be bitter in the presence of DES, highlighting the complex interactions between these compounds and their impact on taste perception.

### Hydration numbers 

The hydration number values (through utilization of Eq. 4) of *GBP* in studied systems has been tabulated within Table 5.

**Table 5:** Hydration numbers (${n_H}$), of GBP in water and in various aqueous choline based ionic liquids solutions at 298 K.

*m* (mol·kg^−1^)	${n_H}$
GBP in aqueous solutions of Betaine	
0.0100	3.556
0.0300	2.546
0.0500	2.271
GBP in aqueous solutions of DES (betaine-urea 1:2)	
0.0100	2.641
0.0300	2.460
0.0500	2.425
GBP in aqueous solutions of IL (betaine octyl ester chloride)	
0.0100	2.954
0.0300	2.706
0.0500	2.612

While the change in volume attributed to electrostriction is related to the number of water molecules associated with GBP, termed the hydration number (*n*_*H*_), accurately quantifying the number of water molecules interacting with solute species remains challenging despite extensive structural and computational investigations. This study determined the hydration numbers using the following equation^51^:

4 $${n_H}=\frac{{V_{\phi }^{0}\left( {elect.} \right)}}{{V_{E}^{0} - V_{B}^{0}}}$$

where $V_{\phi }^{0}\left( {elect.} \right)$ represents the electrostriction partial molar volume resulting from *GBP* hydration. $V_{\phi }^{0}\left( {elect.} \right)$ that can be approximated using the $V_{\phi }^{0}$ of *GBP* and its corresponding intrinsic partial molar volume, $V_{\phi }^{0}$(int.), according to the following formula^52^:

5 $$V_{\phi }^{0}\left( {elect.} \right)=V_{\phi }^{0} - V_{\phi }^{0}(\operatorname{int} .)$$

Where:

6 $$V_{\phi }^{0}(\operatorname{int} .)=\left( {\frac{{0.7}}{{0.634}}} \right).V_{\phi }^{0}(cryst.)$$

7 $$V_{\phi }^{0}(cryst.)=\left( {{\raise0.7ex\hbox{$M$} \!\mathord{\left/ {\vphantom {M {{d_{cryst.}}}}}\right.\kern-0pt}\!\lower0.7ex\hbox{${{d_{cryst.}}}$}}} \right)$$

in which $V_{\phi }^{0}(cryst.)$ represents the crystal molar volume of *GBP* and *M*is its molar mass, 0.7 is the packing density for molecules in organic crystals, and 0.634 is the packing density for random packed spheres^53^. The crystalline density (${d_{cryst.}}$) of GBP provided by the Baranowska et al. is 1.257 g.cm^−3 ^^53^. The electrostriction partial molar volume ($V_{E}^{0} - V_{B}^{0}$) is a crucial parameter in estimating the hydration number. Its values at 298 K were reported as -3.3 cm^3^.mol^−1^, respectively^51,52,54^. Here, $V_{E}^{0}$ water represents the molar volume of electrostricted water, and $V_{B}^{0}$ denotes the molar volume of bulk water. By applying these values to Eq. (5), the hydration numbers for GBP were calculated at various concentrations of the betaine-based compounds in water. As presented in Table 5, a discernible trend is observed wherein the hydration number decreases as the concentration of betaine-based compounds in water increases. This phenomenon can be elucidated by the notion that the interaction between solute molecules intensifies with a rise in the molality of the solute, specifically the GBP^55^. The values of the hydration number (*n*_H_) are documented in Table 5, which clearly indicates a reduction in (*n*_H_ ) values corresponding to higher concentrations of betaine-based compounds. This reduction in hydration number is significant, as it reflects the number of water molecules associated with the hydration of GBP. The observed decrease in (*n*_H_) values with increasing concentrations of betaine-based compounds suggests a corresponding increase in interactions between the solute and cosolute (GBP and betaine-based compounds). Such findings imply that betaine-based compounds exert a dehydration effect on GBP, highlighting the complex interplay between solute concentration and hydration dynamics in aqueous solutions^47,56^.

### Viscosity *B*-coefficients results

The experimental viscosity values (*η*) of gabapentin in aqueous betaine-based compound solutions, with varying concentration ranges of 0.01, 0.03, and 0.05 (mol·kg⁻¹) at a temperature of 298 K, have been tabulated within the Table 6.

**Table 6:** The viscosity (*η*) values of GBP in aqueous Betaine, DES, IL solution in 0.01,0.03,0.05 molality concentration at 298 K.

m_solvent_ (mol kg^−1^)					
Betaine					
0.0101		0.0297		0.0498	
*m* (mol kg^−1^)	$\:\eta\:$ (mPa s^−1^)	*m* (mol kg^−1^)	$\:\eta\:$ (mPa s^−1^)	*m* (mol kg^−1^)	$\:\eta\:$ (mPa s^−1^)
0.00	0.881	0.0000	0.885	0.0000	0.892
0.0010	0.883	0.0010	0.890	0.0009	0.897
0.0049	0.885	0.0049	0.895	0.0049	0.901
0.0100	0.887	0.0099	0.897	0.0097	0.903
0.0201	0.893	0.0204	0.897	0.0200	0.904
0.0293	0.896	0.0298	0.904	0.0294	0.906

DES (betaine-urea 1:2)					
0.0099		0.0298		0.0504	
*m* (mol kg^−1^)	$\:\eta\:$ (mPa s^−1^)	*m* (mol kg^−1^)	$\:\eta\:$ (mPa s^−1^)	*m* (mol kg^−1^)	$\:\eta\:$ (mPa s^−1^)
0.0000	0.883	0.0000	0.889	0.0000	0.897
0.0019	0.887	0.0010	0.894	0.0010	0.905
0.0050	0.892	0.0050	0.898	0.0050	0.907
0.0100	0.898	0.0099	0.901	0.0099	0.909
0.0200	0.904	0.0200	0.906	0.0193	0.911
0.0295	0.908	0.0296	0.91	0.0298	0.915

IL (betaine octyl ester chloride)					
0.0102		0.0301		0.0503	
*m* (mol kg^−1^)	$\:\eta\:$ (mPa s^−1^)	*m* (mol kg^−1^)	$\:\eta\:$ (mPa s^−1^)	*m* (mol kg^−1^)	$\:\eta\:$ (mPa s^−1^)
0.00		0.0000		0.0000	0.8988
0.0010	0.888	0.001	0.897	0.0010	0.921
0.0050	0.89	0.0049	0.901	0.0049	0.925
0.0099	0.894	0.0099	0.904	0.0100	0.929
0.0198	0.9	0.0203	0.91	0.0199	0.937
0.0301	0.91	0.0299	0.915	0.0289	0.927

^a^The standard uncertainties for molality, temperature and pressure were *u* (*m*) *=* 0.001 mol kg^−1^, *u* (*T*) *=* 0.2 K, *u* (*P*) *=* 10.5 hPa, respectively with level of confidence 0.95. The standard combined uncertainty for viscosity was about, *u*_*c*_ (*η*) = 0.02 mPa s^−1^ (level of confidence 0.68).

**Fig. 3:**
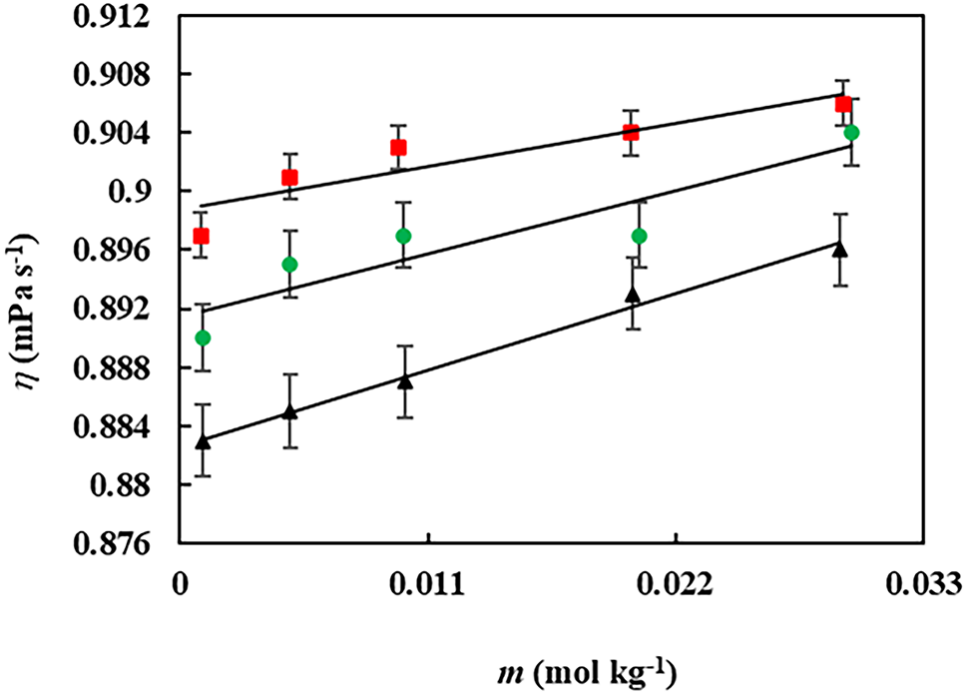
The Viscosity, ($\:\eta$ (mPa s^−1^)), of gabapentin in the presence of various concentrations of aqueous betaine: (▪); 0.05, (●​​); 0.03, (▲); 0.01 mol·kg^−1^ at a temperature of 298 K.

The viscosity plot of gabapentin in the presence of various concentration of aqueous betaine solution has been depicted within the Fig. 3.

The viscosity values of gabapentin in aqueous betaine-based compound solutions demonstrate a positive correlation with increasing concentrations of both gabapentin and the betaine-based compound. As the concentration of either component in the aqueous solution rises, a corresponding increase in solution viscosity is observed^57^. The variation in relative viscosity (*ηr*) of gabapentin in both pure water and aqueous solutions of betaine-based compounds can be effectively modeled using the Jones-Dole equation^58^:

8 $$\frac{\eta }{{{\eta _0}}}=1+A{c^{1/2}}+Bc$$

The Falkenhagen coefficient (*A*) and viscosity *B*-coefficients (*B*) in the aforementioned equation are employed to elucidate solute-solvent interactions^59^. The *B*-coefficient, in particular, has been shown to be a valuable tool in this regard, as it is influenced by factors such as solute size, shape, and charge^60^. The Falkenhagen coefficient was determined using the Least-Square Fitting method and was found to be small and negligible due to the weak solute-solute interactions present in the investigated systems^61^. Consequently, the Falkenhagen coefficient was considered negligible, leading to the simplified Eq. 12^62^:

9 $$\frac{\eta }{{{\eta _0}}}=1+Bc$$

where *η* and *η₀* represent the viscosities of the solutions (gabapentin in aqueous betaine-based compound solutions) and the pure solvent (aqueous betaine-based compound solutions), respectively. The variable *c* denotes the molar concentration of gabapentin in the aqueous betaine-based compound solutions. The viscosity *B*-coefficients were determined from the slope of the linear plot of *η/η₀* versus *c*, obtained through a least-squares regression analysis. The calculated viscosity *B*-coefficients and *A*-coefficients for the studied solutions, derived from fitting the experimental viscosity data to the Jones-Dole equation, have been tabulated within Table 7.

**Table 7:** Second viscosity *B* coefficient (Jones–Dole equation) value for GBP in 0.01, 0.03 and 0.05 molality (mol kg^−1^) concentration of aqueous betaine-based compounds solutions at 298 K.

*m*_solvent_ (mol kg^−1^)	*B* (dm^3/2^ mol^−1/2^)	*σ* (*η*)
Betaine		
0.0099	0.850 ± 0.03	0.01
0.0298	0.842 ± 0.04	0.06
0.0496	0.843 ± 0.09	0.09
DES (betaine-urea 1:2)		
0.0103	1.762 ± 0.13	0.06
0.0300	0.850 ± 0.09	0.07
0.0504	-0.275 ± 0.02	0.01
IL(betaine octyl ester chloride)		
0.0093	2.009 ± 0.31	0.03
0.0306	-0.156 ± 0.06	0.04
0.0493	-2.704 ± 0.13	0.09

^a^The standard uncertainties for molality, temperature and pressure were *u* (*m*) *=* 0.001 mol kg^−1^, *u* (*T*) *=* 0.2 K, *u* (*P*) *=* 10.5 hPa, respectively with level of confidence 0.95. The standard combined uncertainty for viscosity was about, *u*_*c*_ (*η*) = 0.02 mPa s^−1^ (level of confidence 0.68).

The viscosity *B*-coefficient provides valuable insights into the size, shape, charge, and structural effects induced by solute-solvent interactions^63^. This parameter offers a means to assess the solvation behavior of solutes in solution and their influence on the solvent structure in the vicinity of solute molecules^64^. It reflects the net structural effects arising from the interaction of charged end groups, hydrophilic, and hydrophobic groups of the solute with the solvent molecules^65^. The positive values of the viscosity *B*-coefficients for gabapentin in various concentrations of aqueous betaine-based compounds solutions suggest a greater kosmotropic effect of GBP in the aqueous solutions of betaine-based compounds^66^. This further indicates stronger solute-solvent interactions within the studied systems. The viscosity measurements indicated a significant variation in the *B*-coefficient values among the studied systems. The DES exhibited the highest *B*-coefficient values, while the betaine and betaine octyl ester chloride (IL) displayed the lowest. Notably, the *B*-coefficient values for gabapentin in aqueous IL solutions were negative. This observation suggests that the IL may possess desirable properties for enhancing the drug-related characteristics of gabapentin. In the realm of biomedical applications, solutions with negative *B*-coefficient values can potentially improve the delivery of drugs and other therapeutic agents by facilitating their diffusion and transport properties^67^.

### Theoretical framework

**Fig. 4:**
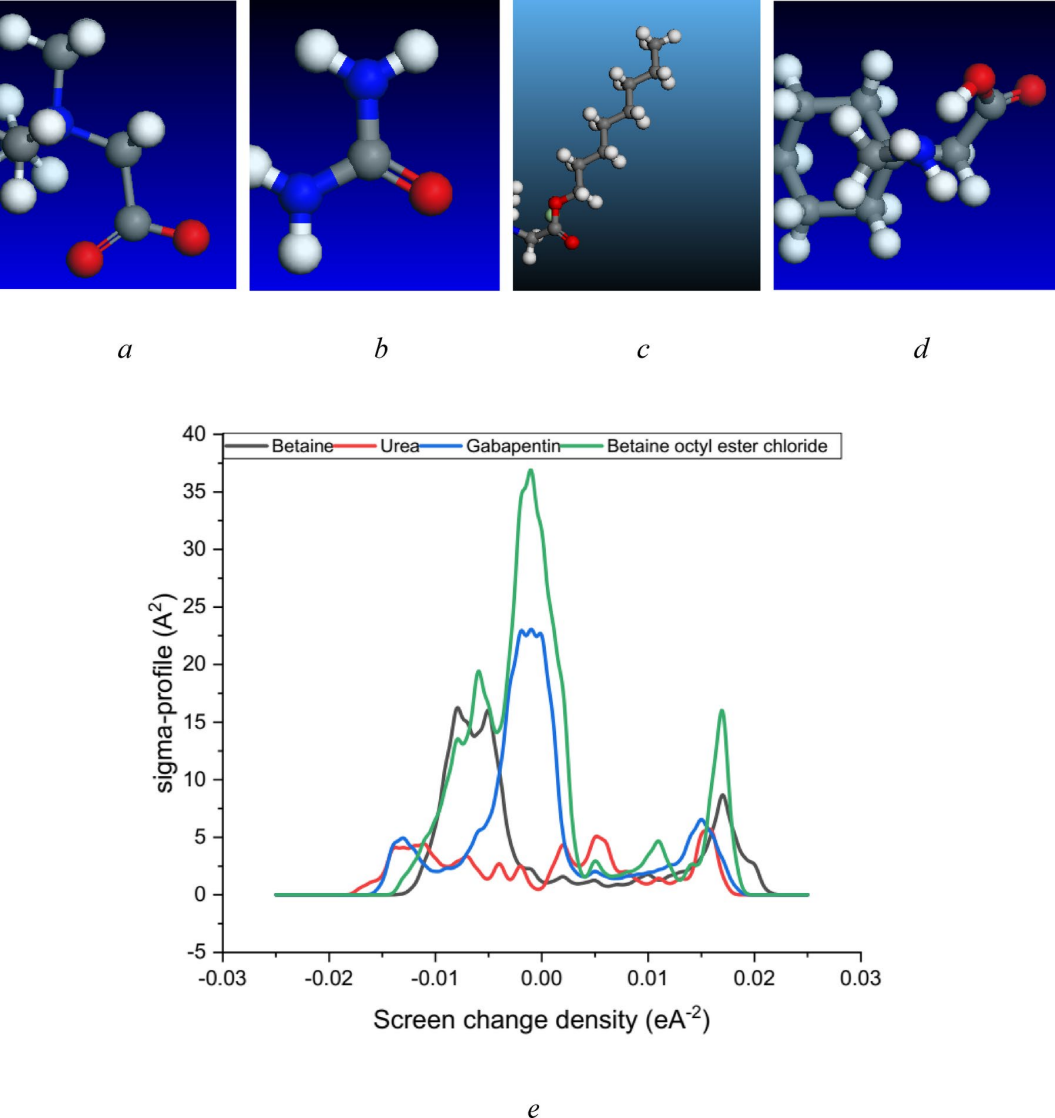
The optimized molecular structure (drawn by Biovia, material studio Dmol3, 2022( and σ-profile of (**a**) Betaine, (**b**) urea, (**c**) Betaine octyl ester chloride, (**d**) Gabapentin, and (**e**) the sigma profile plot obtained from Dmol3 and COSMO result.

The theoretical framework of this study was primarily established through DFT calculations using the Dmol3 module. Figure 4 illustrates the COSMO results, including the *σ*-profile and the optimized molecular structures of the investigated materials.

A fundamental aspect of COSMO-based thermodynamics is the *σ*-profile, a molecular fingerprint that characterizes the surface charge distribution of a molecule^68^. COSMO-based models, such as COSMO-RS and COSMO-SAC, leverage *σ*-profiles to predict thermodynamic properties and intermolecular interactions^69^. Traditionally, *σ*-profiles are derived from computationally intensive density functional theory (DFT) calculations of molecular electron density^70^. The *σ*-profile serves as a powerful tool for analyzing the electronic charge distribution on molecules^71^. It offers valuable insights into molecular polarity, reactivity, and intermolecular interactions. In the context of ionic liquids, *σ*-profiles aid in understanding the charge distribution between the cation and anion, a critical factor in determining their unique physicochemical properties^72^.

By analyzing the *σ*-profile of a molecule, regions of high and low electron density can be identified. These regions are directly correlated with the presence of functional groups, such as polar groups or aromatic rings, which significantly influence the molecule’s reactivity and properties^71,73^. Additionally, σ-profiles can be employed to predict a molecule’s dipole moment and its interactions with other molecules, including solvents and charged species^74^. The *σ*-profile density distributions of betaine, urea, ionic liquids (ILs), and gabapentin, as derived from COSMO analysis using Dmol^3^ has been depicted in the Fig. 4. Additionally, Table 8 presents the calculated cavity surface area (*A*), total cavity surface volume (*V*), dielectric (solvation) energy, highest occupied molecular orbital (HOMO) energy, and lowest unoccupied molecular orbital (LUMO) energy values obtained from COSMO and Dmol^3^ calculations.

**Table 8:** The surface area and total volume of cavity, dielectric (solvation) energy, HOMO and LUMO values obtained from COSMO and Dmol3 calculations.

Chemicals	*A* (Å ^2^)	*V* (Å^3^)	Dielectric (solvation) energy (kcal mol^−1^)	HOMO	LUMO
Gabapentin	198.232	202.454	-26.60	47	48
Betaine	150.791	143.026	-38.77	32	33
Urea	89.216	69.415	-17.72	16	17
Betaine octyl ester chloride	89.228	69.427	-17.68	16	17

The predominant negative charge density observed in most distributions is a characteristic feature of ionic liquids, resulting from the significant charge separation between the cation and anion. The peaks within the σ-profiles correspond to regions of highest electron density^75^. For the studied chemicals, these peaks are situated around σ values of -0.02 to 0.02, indicating a relatively broad distribution of negative charge across the molecular surface. In contrast, gabapentin exhibits a narrower peak centered around − 0.01 to 0.00, suggesting a more localized distribution of negative charge. The height of these peaks correlates with the magnitude of the negative charge density. Betaine octyl ester chloride (ILs) with longer alkyl chains exhibits higher peak intensities compared to shorter-chain urea and betaine, suggesting a greater concentration of negative charge on the longer chains.

The negative charge distribution in both betaine-based compounds and gabapentin can be attributed to the presence of charged head groups^76^. Oxygen atoms within these head groups tend to possess a higher electron density than carbon atoms in the alkyl chains, resulting in a concentration of negative charge in the head group region^77^. The broader peak observed for longer-chain betaine-based compounds indicates a more delocalized negative charge along the alkyl chain, possibly due to increased chain flexibility. In contrast, gabapentin’s narrower peak suggests a more localized negative charge distribution, potentially influenced by the presence of the aromatic ring and hydroxyl group. The negative charge distribution in betaine-based compounds and gabapentin has implications for their properties and interactions with other molecules. For example, the presence of a negative charge can enhance interactions with positively charged surfaces or molecules, such as proteins or nanoparticles^78^. Additionally, the negative charge can influence the solubility of IL in water and other polar solvents.

Also, the related dielectric (solvation) energy and other properties that could be used for the interpretation of hydration behavior of the betaine-based compounds and the drug besides the cavity surface area and volume that has presented in Table 8.

### Surface tension and critical micelle concentration results

Betaine and betaine-based ionic liquids (ILs) have been extensively studied for their surface-active properties. Surfactants, due to their amphiphilic nature, self-assemble into colloidal structures above the critical micelle concentration (CMC), exhibiting unique properties^79^. This study used static surface tension measurements (presented in Table S1) to determine the CMC values of betaine, betaine-urea DES (1:2), and betaine octyl ester chloride ionic liquid.

The experimental surface tension data for betaine, betaine-urea deep eutectic solvent (DES) (1:2 molar ratio), and betaine octyl ester chloride in aqueous gabapentin solutions at concentrations ranging from 0.0000 to 0.0500 mol kg⁻¹ at 298 K are tabulated in Table S1. These measurements were undertaken to elucidate the intermolecular interactions between betaine-based compounds and varying concentrations of gabapentin. A meticulous examination of Table S1 indicates a discernible inverse correlation between the measured surface tension values and the concentration of the aqueous gabapentin solution.

**Fig. 5:**
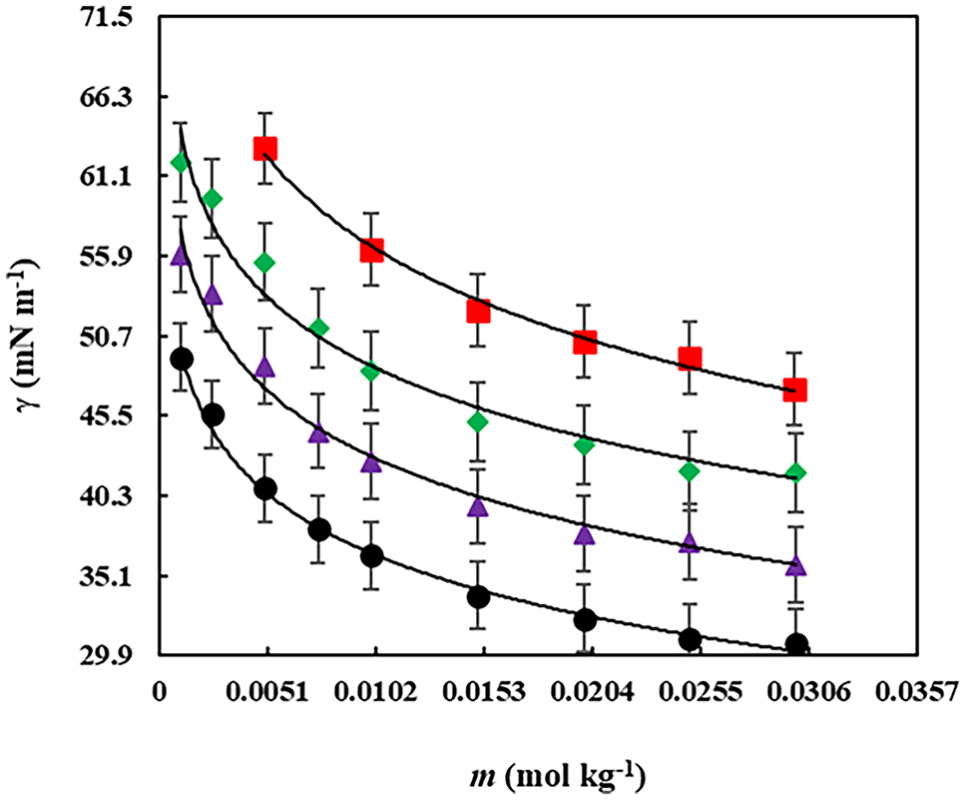
The surface tension (*γ*) of betaine in various molality (mol kg^[-3^) concentrations of aqueous gabapentin solutions at 298 K: ▪, 0.0000; ♦, 0.0100; ▲, 0.0300; and ●, 0.0500.

As depicted in Fig. 5 and Table S1, the measured surface tension decreased with increasing concentrations of betaine-based compounds in both water and aqueous gabapentin solutions. This decrease is attributed to the rapid saturation of the surface by a high number of betaine-based compounds.

As concentration of betaine-based compounds within the aqueous gabapentin solutions increases, these molecules self-assemble into micelles above a critical micelle concentration (CMC). As depicted within the Table 9, the structure of betaine-based compounds influences their CMC, with longer alkyl chains and more hydroxyethyl groups generally leading to lower CMC values.

**Table 9:** The Surface-active parameters of Betaine, DES (betaine + urea), IL (betaine octyl ester chloride) in various molality concentrations of aqueous Gabapentin solutions (from 0.0000 to 0.0500$mol \cdot k{g^{ - 1}}$) at 298 K and ambient pressure.

CMC $\:\left(\text{m}\text{o}\text{l}\:{\text{k}\text{g}}^{-1}\right)$	$\gamma$ (mN m^−1^)	$\:\varPi\:$ (mN m^−1^)	10^3^$\:\times\:{\varGamma\:}_{max}$ $\:\left(\text{m}\text{o}\text{l}\:{\text{m}}^{-2}\right)$	$\:{A}_{min}$ (Å^2^)	$\:{\varDelta\:G}_{mic}$ (kJ mol^−1^)	$\:{\varDelta\:\text{G}}_{\text{a}\text{d}\text{s}}$ (kJ mol^−1^)
Betaine + water						
0.0148	56.3	9.1	2.039	0.081	-23.089	-18.627
		15.7	1.858	0.089	-21.369	-12.917
		19.6	1.705	0.097	-20.372	-8.878
		21.7	1.575	0.105	-19.657	-5.880
		23.2	1.461	0.114	-19.100	-3.222
		24.8	1.363	0.122	-18.657	-0.459
Betaine in 0.0099 mol kg^−1^ concentration of aqueous gabapentin solution						
0.0100	48.4	10.0	-0.347	-0.478	-26.916	-55.716
		12.4	1.109	0.15	-24.899	-13.717
		16.5	1.734	0.096	-23.136	-13.618
		20.8	1.815	0.092	-22.099	-10.638
		23.6	1.748	0.095	-21.382	-7.881
		26.9	1.486	0.112	-20.383	-2.286
		28.4	1.165	0.143	-19.638	4.742
		30.1	0.871	0.191	-19.111	15.433
		30.2	0.548	0.303	-18.618	36.527
Betaine in 0.0299 mol kg^−1^ concentration of aqueous gabapentin solution						
0.0075	45.4	16.0	-0.163	-1.022	-27.101	-125.56
		18.6	1.193	0.139	-24.849	-9.257
		23.3	1.652	0.100	-23.147	-9.047
		27.5	1.692	0.098	-22.128	-5.873
		29.5	1.605	0.103	-21.362	-2.9801
		32.4	1.367	0.121	-20.437	3.265
		34.2	1.066	0.156	-19.684	12.4
		34.7	0.783	0.212	-19.130	25.186
		36.2	0.513	0.324	-18.677	51.857
Betaine in 0.0498 mol.kg^−1^ concentration of aqueous gabapentin solution						
0.0065	40.7	22.7	0.530	0.313	-21.228	21.571
		26.4	1.159	0.143	-24.918	-2.142
		31.2	1.364	0.122	-23.155	-0.280
		33.9	1.356	0.122	-22.118	2.875
		35.6	1.296	0.128	-21.402	6.078
		38.2	1.132	0.147	-20.402	13.332
		39.7	0.955	0.174	-19.657	21.893
		41.0	0.799	0.208	-19.130	32.180
		41.3	0.631	0.263	-18.637	46.823
DES (betaine-urea 1:2) + water						
0.0150	66.9	13.0	-0.010	-16.116	-27.077	-1288.703
		8.6	1.976	0.084	-24.801	-20.450
		7.9	1.831	0.091	-23.087	-18.772
		6.9	1.570	0.106	-22.040	-17.644
		5.6	1.567	0.106	-21.368	-17.795
		5.1	2.147	0.077	-20.372	-17.997
		6.2	3.251	0.051	-19.660	-17.753
		7.0	4.689	0.035	-19.100	-17.608
		10.9	6.364	0.026	-18.634	-16.921
DES (betaine-urea 1:2) in 0.0103 mol.kg^−1^ concentration of aqueous gabapentin solution						
0.0100	53.4	26.1	0.008	19.799	-27.102	3084.853
		21.6	0.055	2.992	-24.825	364.368
		19.4	0.328	0.506	-23.111	36.035
		18.9	0.443	0.375	-22.112	20.535
		18.6	0.530	0.313	-21.375	13.692
		18.3	0.733	0.227	-20.370	4.608
		19.4	1.012	0.164	-19.660	-0.484
		19.9	1.363	0.122	-19.105	-4.502
		21.4	1.781	0.093	-18.64	-6.624
DES (betaine-urea 1:2) in 0.0297 mol kg^−1^ concentration of aqueous gabapentin solution						
0.0075	49.8	28.8	4.014	0.041	-27.092	-19.917
		24.5	2.539	0.065	-24.823	-15.173
		23.0	1.698	0.098	-23.117	-9.570
		22.2	1.318	0.126	-22.129	-5.281
		21.6	1.086	0.153	-21.402	-1.511
		22.8	0.828	0.201	-20.364	7.174
		21.3	0.709	0.234	-19.712	10.313
		23.0	0.633	0.262	-19.139	17.199
		23.2	0.592	0.281	-18.693	20.529
DES (betaine-urea 1:2) in 0.0501 mol kg^−1^ concentration of aqueous gabapentin solution						
0.0050	46.2	31.9	1.654	0.100	-27.104	-7.812
		27.8	-1.109	-0.150	-24.807	-49.881
		25.0	-0.065	-2.560	-23.07	-408.452
		25.0	0.170	0.978	-22.114	125.137
		25.1	0.118	1.413	-21.379	192.134
		26.0	0.008	20.728	-20.362	3225.001
		25.7	0.249	0.668	-19.682	83.647
		26.4	0.947	0.175	-19.099	8.766
		28.8	1.989	0.083	-18.643	-4.162
IL (betaine octyl ester chloride) + water						
0.0125	28.4	27.0	0.002	91.804	-27.077	14899.651
		32.4	0.616	0.269	-24.801	27.761
		38.7	1.064	0.156	-23.087	13.29
		43.4	0.912	0.182	-22.04	25.531
		45.6	0.660	0.252	-21.368	47.742
		48.1	0.269	0.618	-20.372	158.748
		50.4	0.136	1.219	-19.660	350.437
		51.6	0.247	0.673	-19.100	190.116
		52.7	0.558	0.297	-18.634	75.778
IL(betaine octyl ester chloride) in 0.0105 mol kg^−1^ concentration of aqueous gabapentin solution						
0.0125	22.6	29.5	0.034	4.889	-27.174	841.402
		35.6	0.193	0.860	-24.854	159.45
		42.2	0.445	0.373	-23.087	71.740
		45.9	0.356	0.467	-22.095	106.944
		48.7	0.177	0.938	-21.332	253.853
		51.3	-0.058	-2.866	-20.413	-905.853
		52.8	-0.190	-0.875	-19.654	-297.992
		54.0	-0.176	-0.945	-19.102	-326.267
		55.0	-0.057	-2.899	-18.661	-978.869
IL(betaine octyl ester chloride) in 0.0304 mol kg^−1^ concentration of aqueous gabapentin solution						
0.0094	21.9	33.6	2.727	0.061	-26.983	-14.664
		40.7	0.905	0.183	-24.753	20.201
		46.6	1.182	0.140	-23.079	16.331
		49.6	0.901	0.184	-22.143	32.925
		51.6	0.692	0.240	-21.306	53.236
		53.2	0.588	0.282	-20.436	70.005
		54.6	0.595	0.279	-19.711	72.109
		55.5	0.468	0.355	-19.061	99.510
		55.9	0.101	1.638	-18.66	532.678
IL(betaine octyl ester chloride) in 0.0499 mol kg^−1^ concentration of aqueous gabapentin solution						
0.0073	20.7	37.0	0.801	0.207	-27.079	19.114
		44.9	1.243	0.134	-24.807	11.303
		50.7	0.905	0.183	-23.089	32.909
		52.7	0.679	0.245	-22.084	55.511
		54.3	0.545	0.305	-21.371	78.244
		55.8	0.407	0.408	-20.367	116.738
		56.4	0.339	0.490	-19.654	146.786
		56.9	0.293	0.567	-19.101	175.163
		57.4	0.252	0.660	-18.649	209.362

^a^The molality of the prepared aqueous gabapentin solution (solvent). The standard uncertainties for molality, temperature and pressure were *u* (*C*) = 0.001 mol m^−3^, *u* (*T*) = 0.5 K, and u(*P*) = 0.01 MPa respectively with level of confidence 0.95. The standard combined uncertainty for surface tension were about, *uc* (*σ*) = 0.01 mN·m^−1^ (level of confidence 0.68), respectively.

Additionally the relevant surface-active properties of interface surface pressure ($\Pi$), surface tension of the CMC point (${\gamma _{CMC}}$), minimum surface area occupied per molecule (${A_{\hbox{min} }}$), Gibbs maximum excess surface concentration (${\Gamma _{\hbox{max} }}$) were computed from measured surface tension data and presented in Table 9. The $\Pi$, has been used as an illustrator to show the difference between surface tension of the pure solvent and the surface tension of betaine-based compounds, and can be computed through following expression:

11 $$\Pi ={\gamma _0} - \gamma$$

Here ${\gamma _0}$, is the surface tension of pure solvent (water). The ${\Gamma _{\hbox{max} }}$, is a parameter related to describing the surface concentration and it is defined through the following expression^80^:

12 $${\Gamma _{\hbox{max} }}= - \frac{1}{{nRT}}\left[ {\frac{{\partial \sigma }}{{\partial \ln C}}} \right]$$

where, *n* is the number of ionic spices resulted of the dissociation of spices in water which in our case is the equivalent of one, *R*, is the gas constant, *T*, is the absolute temperature and *C*, is the concentration of Betaine based compounds in the solution. Table 9 presents the ${\Gamma _{\hbox{max} }}$ values for the studied Betaine based compounds in various concentrations of gabapentin in aqueous solutions. The Gibbs maximum excess surface concentration ${\Gamma _{\hbox{max} }}$, a measure of a surfactant’s efficiency and effectiveness in reducing surface tension and forming a monolayer at the air-water interface, was determined for betaine, betaine-urea deep eutectic solvent (DES) (1:2 molar ratio), and betaine octyl ester chloride ionic liquid (IL) in water and various aqueous gabapentin solutions (0.01, 0.03 and 0.05 mol kg^−1^). As it can be seen from Table 9, the related values of the ${\Gamma _{\hbox{max} }}$ decreases as the concentration of the aqueous gabapentin solution’s increases, this observation in the ${\Gamma _{\hbox{max} }}$ values can be attributed to the improved efficiency of the Betaine based compounds at the air-water interface. The lower ${\Gamma _{\hbox{max} }}$ values in the presence of the various concentration of gabapentin aqueous solution, indicate a decrease in the packing of Betaine based compounds molecules at the air/water interface.

Among these compounds, the DES exhibited the highest Gibbs maximum excess surface concentration ${\Gamma _{\hbox{max} }}$, while the IL displayed the lowest. Interestingly, the surface tension measurements (tabulated within Table S1) revealed that the IL had the lowest surface tension, suggesting that it rapidly lowers the surface tension of water, agglomerates within the bulk, and forms micelles more quickly. Conversely, the DES exhibited the highest surface tension. When comparing the three betaine-based compounds based solely on surface tension, the IL would be ranked first, followed by betaine, and then the DES. The reason for the DES’s slight influence on water’s surface tension can be attributed to the fundamental principles of surface tension. Surface tension arises from the cohesive forces between water molecules at the surface, specifically hydrogen bonds. To reduce surface tension, a substance must disrupt these hydrogen bonds. While betaine and the IL can effectively weaken these bonds, the DES, composed of a hydrogen donor and acceptor, tends to strengthen them instead. This is because the DES’s inherent hydrogen bonding nature reinforces the existing hydrogen bonds in water.

The betaine octyl ester chloride ionic liquid (IL) shows significant potential for enhancing gabapentin’s properties in the gastrointestinal tract. Its low Critical Micelle Concentration (CMC) allows it to form micelles at lower concentrations, improving gabapentin’s solubility. The IL’s strong surface activity facilitates effective interaction at the water-air interface, promoting an environment that enhances solubility and dissolution^81^. By forming micelles, the ionic liquid can encapsulate the hydrophobic parts of gabapentin, improving its solubility in the aqueous gastrointestinal environment, leading to better absorption. This ability to rapidly lower surface tension and form micelles makes the IL the most promising candidate for improving drug solubility, dissolution rate, and bioavailability. Consequently, these properties significantly enhance gabapentin’s bioavailability, improving its overall therapeutic effectiveness. In contrast, the DES, while exhibiting unique hydrogen bonding properties, might face challenges in enhancing gabapentin’s properties due to potential competitive interactions between gabapentin and urea with water molecules. Betaine, although less effective than the IL, may still offer some enhancement due to its surface-active properties.

The ${A_{\hbox{min} }}$or the minimum surface area occupied per betaine-based compounds molecule can be computed by utilization of the ${\Gamma _{\hbox{max} }}$values which has been expressed as following expression^82^:

13 $${A_{\hbox{min} }}=\frac{{{{10}^{20}}}}{{{N_A} \cdot {\Gamma _{\hbox{max} }}}}$$

*N*_A_ is the Avogadro number. Also ${A_{\hbox{min} }}$ illustrates the interface packing of the compactness of the betaine-based compounds. The *A*_min_ parameter, provides insights into a molecule’s propensity to form a new surface at the water-air interface. A lower *A*_min_value indicates a stronger tendency for the molecule to agglomerate within the bulk phase rather than forming a surface layer^83^.

The values of ${A_{\hbox{min} }}$ have been also presented in Table 9, through a careful examination of Table 9, a rising trend for ${A_{\hbox{min} }}$values as the concentration of the gabapentin in aqueous solution increased was observed. At the interface of water / air, Betaine based compounds molecules adsorb with their alkyl chains oriented toward the air, which would cause them to have minimum contact with the aqueous phase. Upon analyzing the *A*_min_ values for betaine, DES, and IL, it was observed that betaine and *IL* exhibited the lowest values. This suggests that these compounds prefer to aggregate within the bulk solution rather than forming a new surface at the water-air interface. In contrast, the DES displayed higher *A*_min_ values, indicating a greater tendency to form a surface layer.

The related thermodynamic properties of micellization for the studied systems has been expressed by the standard free energy of micellization *ΔG*_mic_, and standard Gibbs free energy of adsorption $\Delta G_{{ad}}^{0}$, that can be computed from the following equation^84^:

14 $$\Delta {G_{mic}}=RTln{X_{cmc}}$$

15 $$\Delta G_{{ad}}^{0}=\Delta G_{{mic}}^{0} - \frac{\Pi }{{{\Gamma _{\hbox{max} }}}}$$

In the above-cited expressions, ${X_{cmc}}$ illustrates the mole fractional concentration of the employed additives. Table 9 depict the evaluated *ΔG*_mic_, and $\Delta G_{{ad}}^{0}$, for the current studied systems. The thermodynamic analysis of micellization, based on the data presented in Table 9, indicates that the process is spontaneous for all studied betaine-based compounds, as evidenced by the negative Gibbs free energy of micellization (*ΔG*_mic_) values. The addition of gabapentin further promotes micellization, as indicated by more negative *ΔG*_mic_ values. Notably, the most negative *ΔG*_mic_ values are observed for betaine and ionic liquids (ILs) in aqueous gabapentin solutions compared to deep eutectic solvents (DES), suggesting that ILs and betaine may be more effective enhancers of gabapentin’s drug-related properties.

A comparative analysis of *ΔG*_mic_ and the standard Gibbs free energy of adsorption ($\Delta G_{{ad}}^{0}$) provides additional insights. While ILs and betaine exhibit positive $\Delta G_{{ad}}^{0}$ values, indicating a preference for micellization, DES predominantly shows negative $\Delta G_{{ad}}^{0}$ values, suggesting a preference for adsorption. From a thermodynamic standpoint, these findings suggest that micellization is the preferred process for ILs, followed by betaine. Furthermore, the higher positive $\Delta G_{{ad}}^{0}$ values for ILs and betaine compared to DES imply that ILs, followed by betaine, may be more suitable candidates for enhancing gabapentin’s drug-related properties within the gastrointestinal tract. This is likely due to their stronger tendency to form micelles and incorporate drug molecules, potentially leading to improved drug solubility, dissolution rate, and bioavailability. The values of $G_{{\hbox{min} }}^{s}$, (free energy of the surface at equilibrium), for the studied systems has been tabulated in the Table 9.

The study found that GBP content with the solutions reduced the critical micelle concentration (CMC) of betaine-based compounds compared to pure water, suggesting faster surface saturation of the betaine-based compounds. As GBP concentration increased, surface tension decreased and the CMC of betaine-based compounds also declined. This effect is due to the accumulation of gabapentin molecules, which disrupt the favorable interactions between water and the hydrophilic groups of the betaine compounds, leading to accelerated aggregation of the surfactant molecules^85,86^. Consequently, fewer free surfactant molecules are available, lowering the overall CMC. This indicates that higher gabapentin concentrations promote micelle formation at lower surfactant concentrations.

Among the betaine-based compounds investigated, the betaine octyl ester chloride ionic liquid system demonstrated the most pronounced surface activity, as evidenced by its lowest critical micelle concentration (CMC) value. This was followed by the betaine system and, subsequently, the deep eutectic solvent (DES). The observed trend in CMC values, with the betaine octyl ester chloride ionic liquid exhibiting the lowest CMC, suggests that this ionic liquid has the potential to enhance the properties of the gabapentin drug within the gastrointestinal tract.

## Conclusion

An investigation was conducted to explore the interactions between gabapentin (GBP) and three betaine-based compounds: betaine, deep eutectic solvent (DES) composed of betaine and urea in a 1:2 molar ratio, and betaine octyl ester chloride ionic liquid (IL). To achieve this, volumetric, viscosity, and static surface tension techniques were employed in aqueous media. Apparent molar volumes ${V_\varphi }$ of gabapentin in aqueous solutions of the betaine-based compounds were determined from density measurements. Subsequently, standard partial molar properties were derived from these apparent molar volumes. The results of this study indicated that the interactions between gabapentin (GBP) and the betaine-based compounds intensified as the concentration of the betaine-based compounds increased. The apparent specific volume (ASV) of the GBP in the presence of the betaine-based compounds were investigated the DES showed the most ASV number indicating that DES further accommodate the bitter taste of the GBP. The hydration number of GBP was calculated in the studied systems.

The viscosity measurements indicated a significant variation in the viscosity *B*-coefficient values among the studied systems. The DES exhibited the highest *B*-coefficient values, while the betaine and betaine octyl ester chloride (IL) displayed the lowest. Notably, the *B*-coefficient values for GBP in aqueous IL solutions were negative. This observation suggests that the betaine octyl ester chloride ionic liquid may possess desirable properties for enhancing the drug-related characteristics of gabapentin. Surface tension measurements were employed to determine the CMC. Additionally, the influence of gabapentin on the CMC shift was examined at different drug concentrations. Subsequently, the Gibbs free energy of micellization was calculated based on the CMC values to evaluate the thermodynamic parameters associated with micelle formation. The results revealed a decrease in the CMC of betaine-based compounds in the presence of gabapentin, suggesting interactions between the drug and the compounds. Notably, the betaine octyl ester chloride ionic liquid (IL) exhibited the lowest CMC among the studied systems. This finding indicates a stronger propensity for micelle formation at lower concentrations for the IL compared to the other two compounds.

Furthermore, the surface tension measurements demonstrated that the IL possessed the lowest surface tension at all investigated concentrations. Conversely, the DES exhibited the highest surface tension, indicating a weaker interaction with the aqueous environment. Consequently, based solely on surface tension measurements, the ranking for ability to lower surface tension would be IL > betaine > DES. The efficient surface tension reduction and accelerated micelle formation exhibited by the betaine octyl ester chloride ionic liquid suggest its potential as a promising candidate for enhancing gabapentin drug related properties.

COSMO calculations were performed to determine the *σ*-profiles of these molecules, which provide valuable insights into their charge distribution and intermolecular interactions. The *σ*-profiles revealed that betaine-based compounds and gabapentin exhibit a predominantly negative charge distribution, particularly around oxygen atoms in the head groups. Longer-chain betaine-based compounds, such as betaine octyl ester chloride, displayed broader and more intense negative charge distributions due to increased chain flexibility. The negative charge distribution in these molecules can influence their interactions with other molecules, including water and drug molecules, potentially impacting their solubility, micellization behavior, and drug delivery properties. Additionally, the calculated surface cavity area, surface cavity volume, dielectric energy, HOMO, and LUMO energies provide further insights into the molecular properties of these compounds and their potential interactions with water and other molecules. These computational results can help to elucidate the mechanisms underlying the observed experimental behavior of these compounds and guide future research efforts in the development of novel drug delivery systems.

Future research endeavors should delve deeper into the intricate mechanisms underlying the interaction between betaine-based compounds and gabapentin. By expanding the scope of research in in vivo and in vitro studies, significant advancements can be made in improving the therapeutic efficacy and patient compliance associated with gabapentin administration.

## Electronic supplementary material

Below is the link to the electronic supplementary material.

Supplementary Material 1

## Data Availability

The authors confirm that the data supporting the findings of this study are available within the manuscript, figures, tables and supporting information files.
