# Supplementary material for: Gabapentin drug interactions in water and aqueous solutions of green betaine based compounds through volumetric, viscometric and interfacial properties
Source: Sci Rep. 2025 May 14;15:16813. doi: 10.1038/s41598-025-99596-3 (PMC12078619; doi:10.1038/s41598-025-99596-3)
Supplement: Supplementary file 1 — Supplementary Material 1 [file 41598_2025_99596_MOESM1_ESM.docx]

Supplementary Material

**Gabapentin Drug Interactions in Water and Aqueous Solutions of Green Betaine Based Compounds  Through  Volumetric, Viscometric and Interfacial Properties**

Elaheh Janbezar ^a^, Hemayat Shekaari^[[1]](#footnote-1)^* ^a^, Mohammad Bagheri ^a^

*Department of Physical Chemistry, Faculty of Chemistry, University of Tabriz, Tabriz, 5166616471, Iran*

* *Corresponding author. Tel.: +*984133393094. Fax: +984133340191.

E-mail address: [hemayatt@yahoo.com](mailto:hemayatt@yahoo.com) (H. Shekaari).


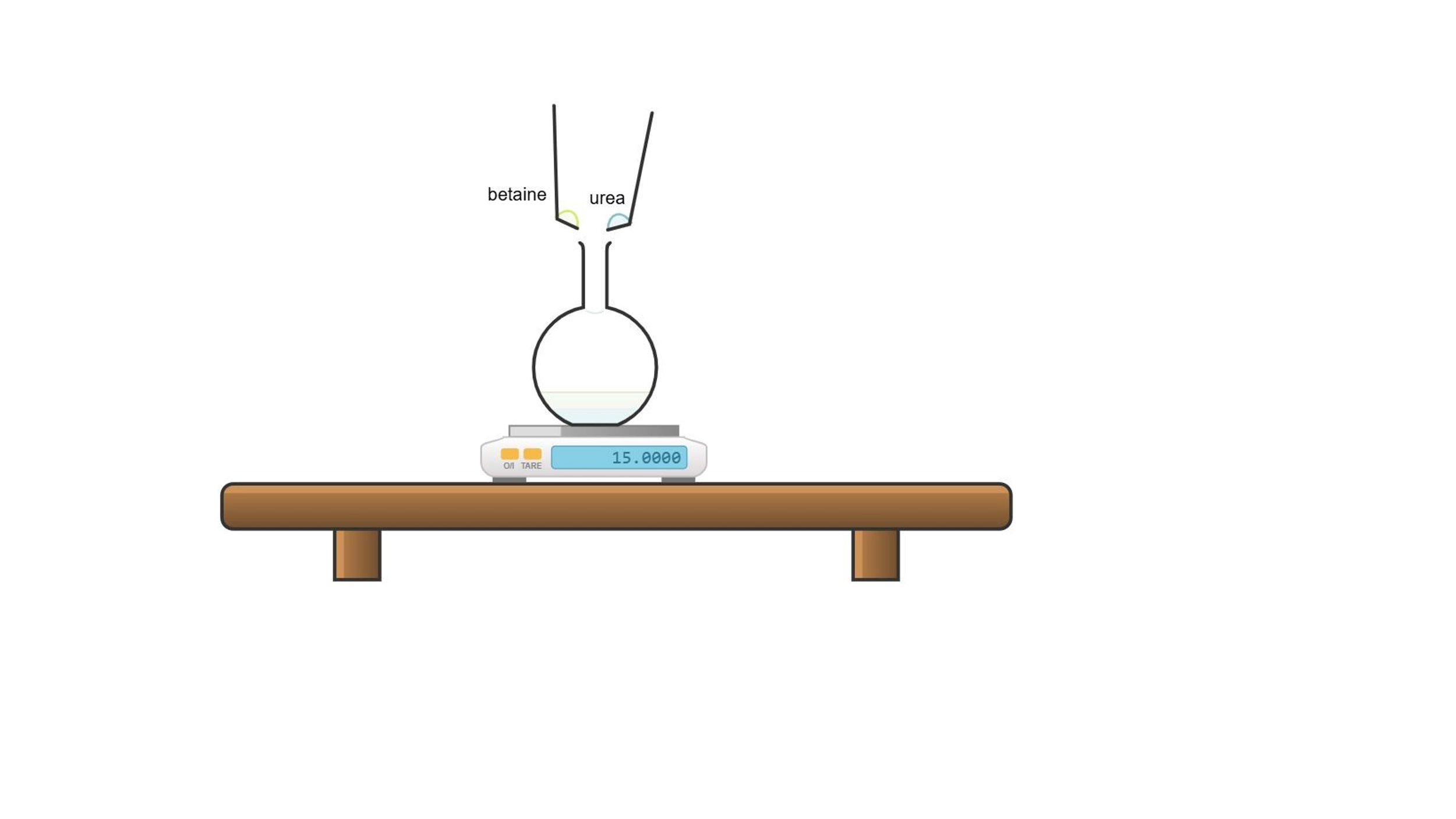
 **
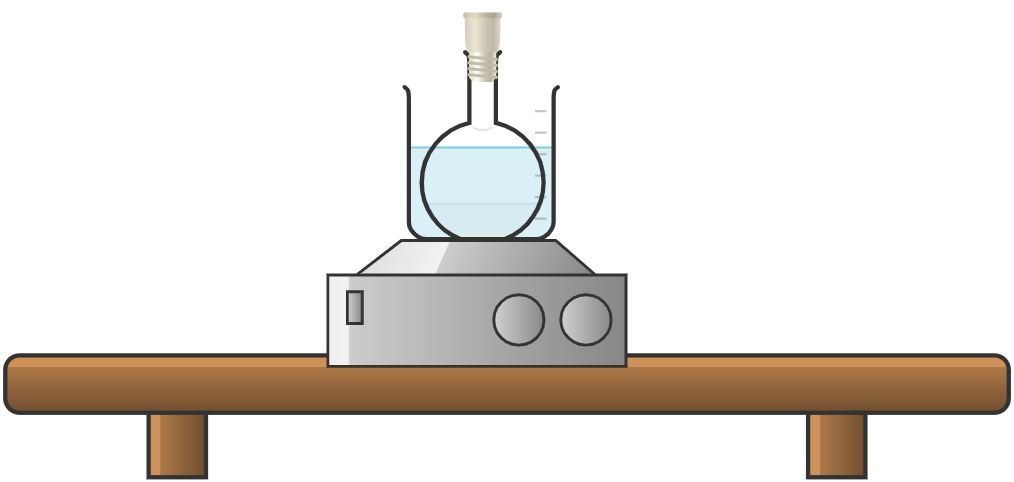
**

Stirred at 80 C^°^ for 2h

**
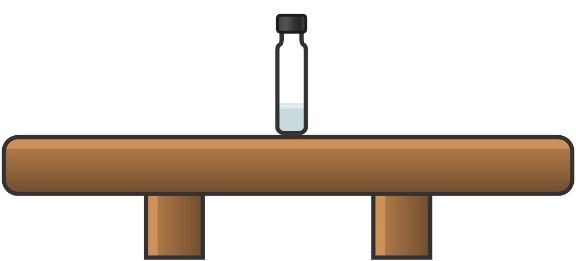
**

Product prepared

**Fig S1.** The synthesis route of the deep eutectic solvent (DES) betaine-urea (1:2 molar ratio)

The Synthesis route of the DES was as follows: a) betaine and urea with (1:2) molar ratio was measured and added to flask, and then the inner content of the flask was stirred at 353.15 K for 2h c) solid product prepared and ready for use.


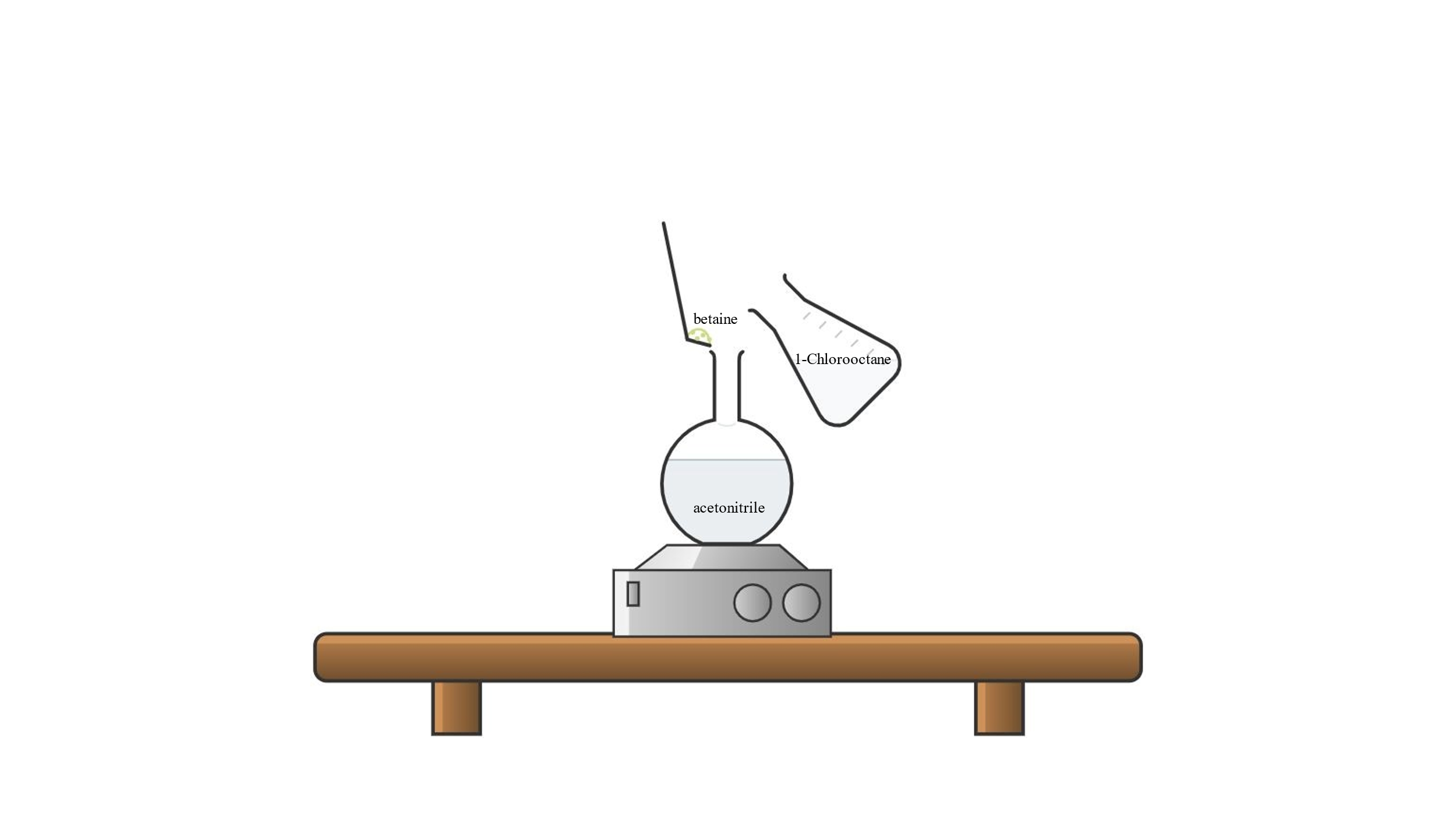
 **
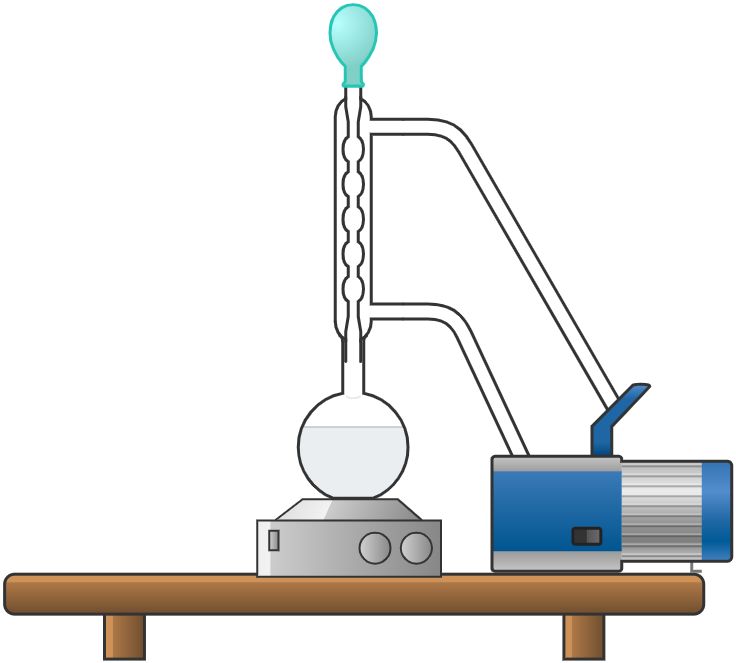
**

a

b

Rotary for remove the solvent and extra alkyl halide

stirred at 353.15 K for 72h

**
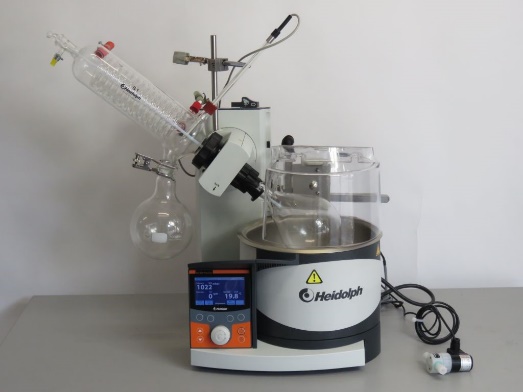
**
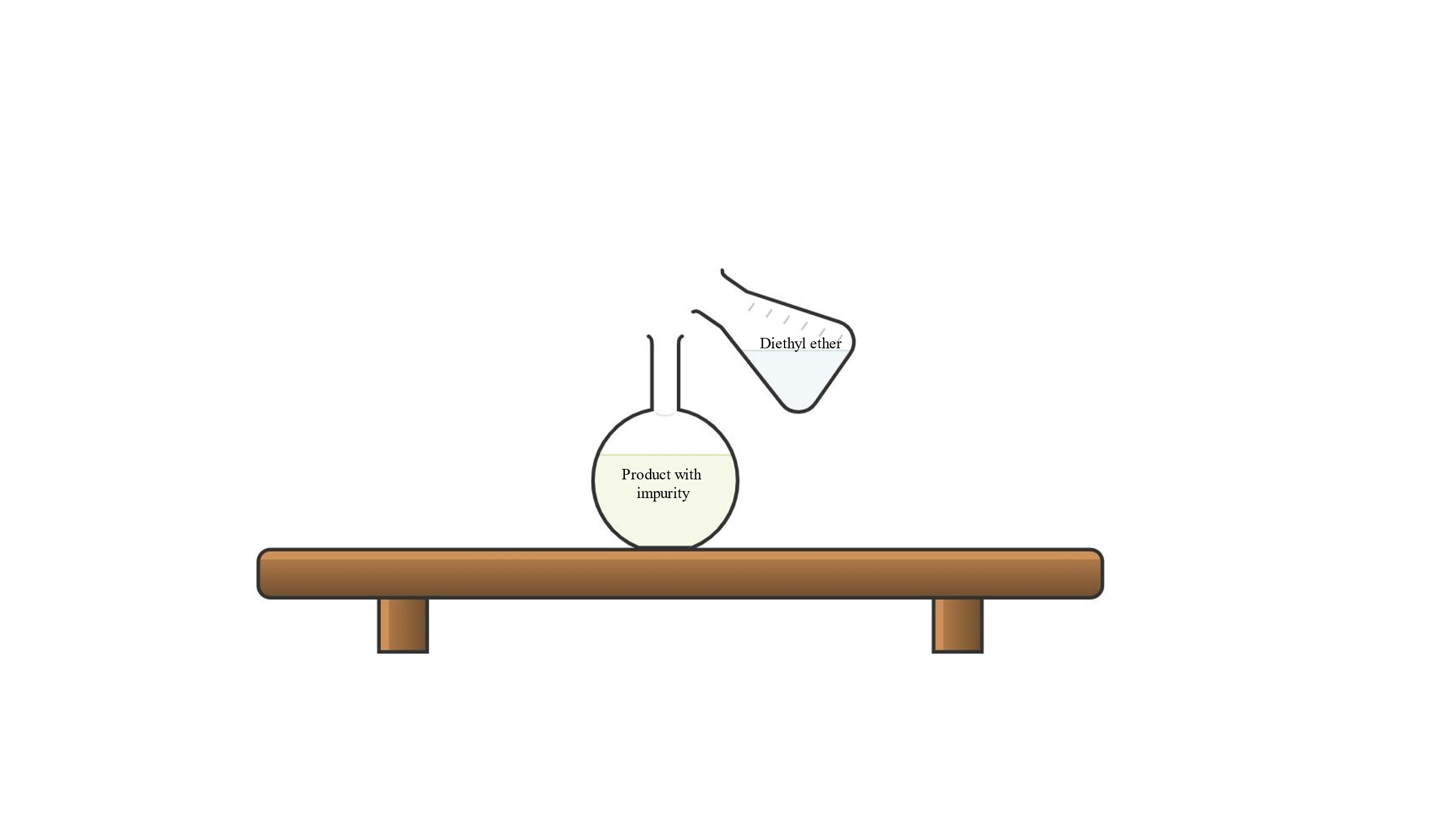


c

d

eliminate the unreacted alkyl halide


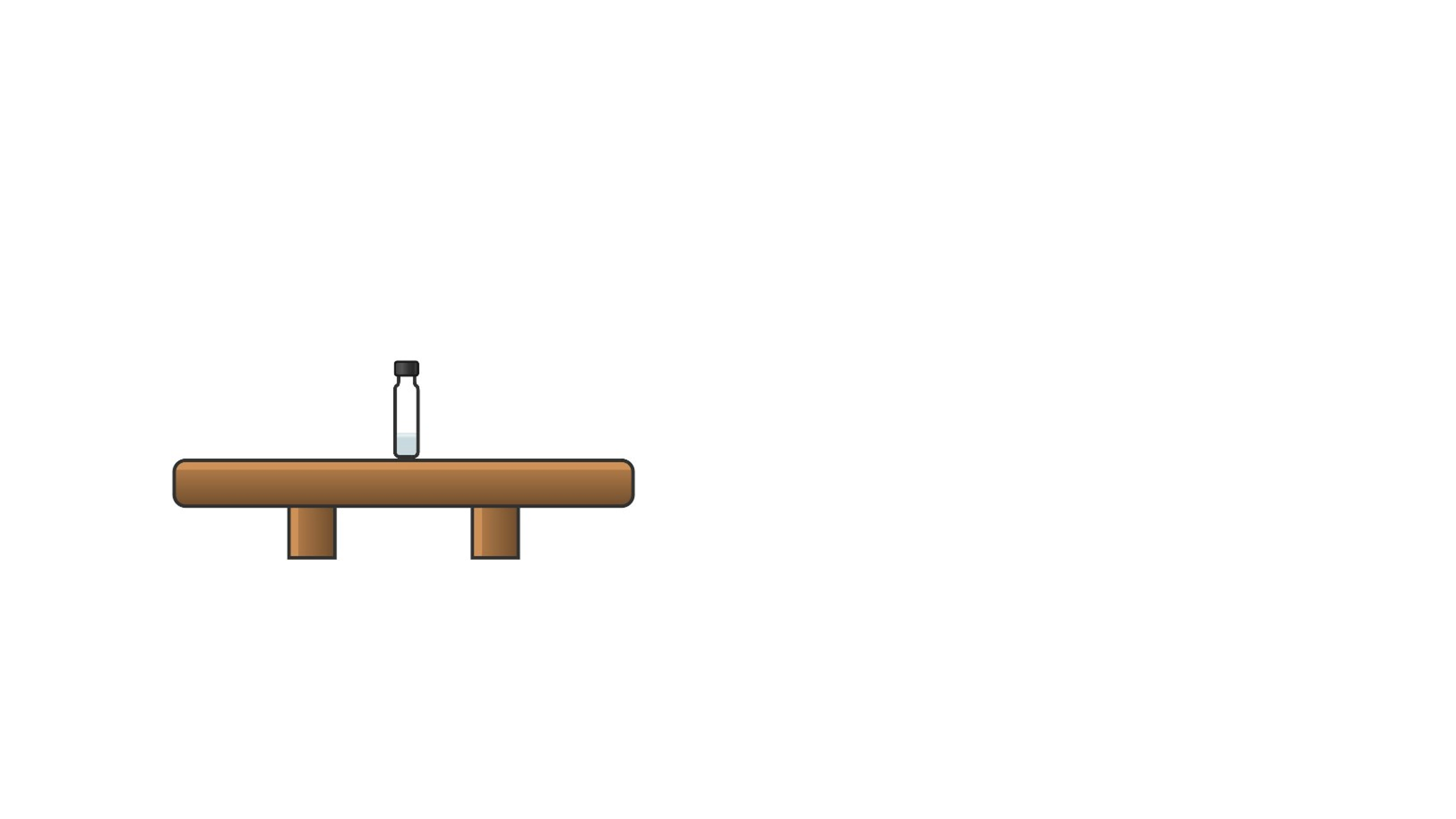


e

Product prepared

**Fig S2.** The synthesis route of the betaine octyl ester chloride ionic liquid (IL)

synthesis of betaine octyl ester chloride: a) betaine and alkyl halide with(1:1.2)molar ratio were added to flask wich containe 50 ml acetonitril b) flask was stirred at 353.15 K for 72h under reflux c) to remove the solvent and extra alkyl halide rotary was used d) diethyl ether was added to precipitate the product and eliminate the unreacted alkyl halide e) solid precipitates were dried and prepared.

**Fig S3.** FT-IR spectrum of betaine octyl ester chloride.

FT-IR spectroscopy was employed to elucidate the functional groups present in the synthesized betaine octyl ester chloride IL (Fig S3). The IR spectrum exhibits several characteristic bands. The broad absorption band centered around 3384 cm⁻¹ is likely due to the N-H stretching vibration of the ammonium group. The strong peak at 1622 cm⁻¹ is characteristic of the asymmetric stretching vibration of the carboxylate group (COO⁻). The peak at 1394 cm⁻¹ is associated with the symmetric stretching vibration of the carboxylate group. The peak at 1332 cm⁻¹ could be attributed to the C-N stretching vibration in the ammonium group or the C-H bending vibration of the alkyl chain. The peak at 893 cm⁻¹ might be related to the C-Cl stretching vibration or a specific bending vibration of the alkyl chain.


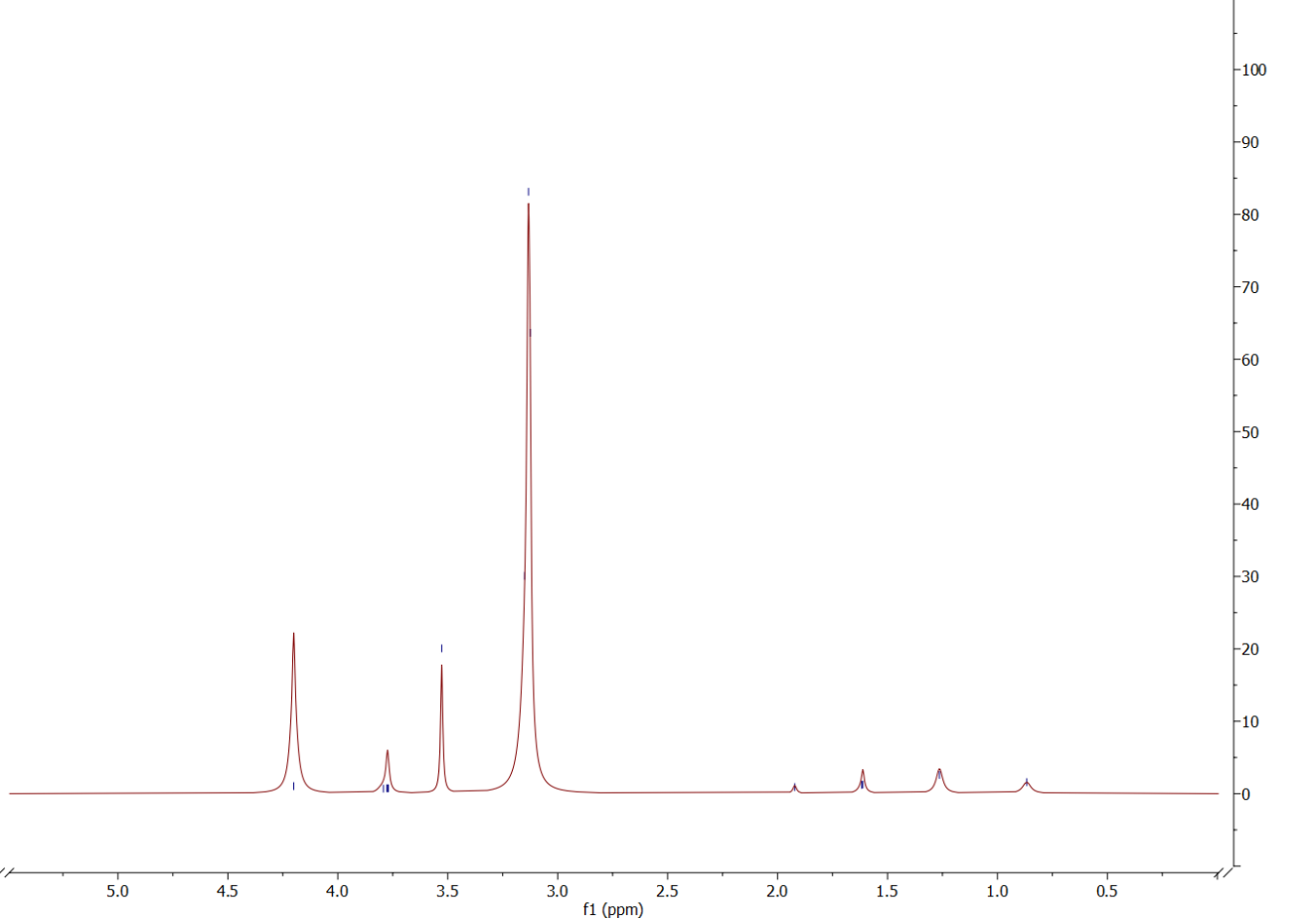


**Fig S4.** ^1^H-NMR spectrum of betaine octyl ester chloride.

^1^H NMR (400 MHz, DMSO) δ 4.20 (s, 4H), 3.82 – 3.75 (m, 1H), 3.53 (s, 1H), 3.13 (d, J = 3.2 Hz, 15H), 1.92 (s, 0H), 1.65 – 1.57 (m, 1H), 1.26 (s, 1H), 0.87 (s, 1H).


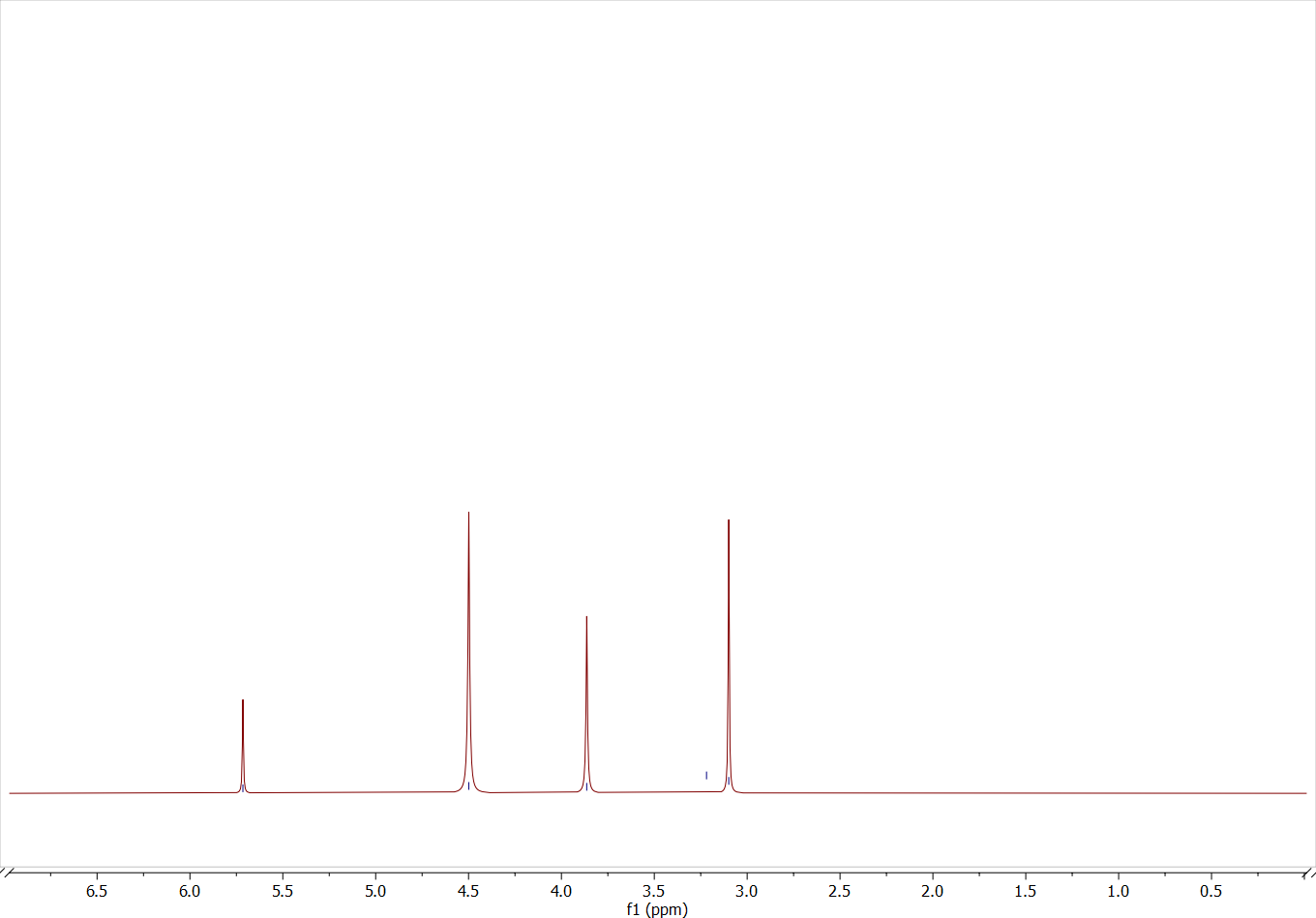


**Fig S5.** ^1^H-NMR spectrum of DES (betaine-urea (1:2)).

^1^H NMR (400 MHz, DMSO) δ 5.71 (s, 2H), 4.50 (s, 6H), 3.86 (s, 3H), 3.10 (s, 4H).

1. [↑](#footnote-ref-1)
